# The Metabolomic Profile in Amyotrophic Lateral Sclerosis Changes According to the Progression of the Disease: An Exploratory Study

**DOI:** 10.3390/metabo12090837

**Published:** 2022-09-04

**Authors:** Carmen Marino, Manuela Grimaldi, Eduardo Maria Sommella, Tania Ciaglia, Angelo Santoro, Michela Buonocore, Emanuela Salviati, Francesca Trojsi, Arianna Polverino, Pierpaolo Sorrentino, Giuseppe Sorrentino, Pietro Campiglia, Anna Maria D’Ursi

**Affiliations:** 1 PhD Program in Drug Discovery and Development, Department of Pharmacy, University of Salerno, Via Giovanni Paolo II, 132, Fisciano, 84084 Salerno, Italy; 2 Department of Pharmacy, University of Salerno, Via Giovanni Paolo II, 132, Fisciano, 84084 Salerno, Italy; 3 Department of Advanced Medical and Surgical Sciences, University of Campania Luigi Vanvitelli, Via Maggiore Salvatore Arena, Contrada San Benedetto, 81100 Caserta, Italy; 4 Institute of Diagnosis and Treatment Hermitage Capodimonte, Cupa delle Tozzole, 2, 80131 Naples, Italy; 5 Institute of Applied Sciences and Intelligent Systems of National Research Council, Via Campi Flegrei 34, 80078 Pozzuoli, Italy; 6 Institut de Neurosciences des Systèmes, Aix-Marseille Université, 13284 Marseille, France; 7 Department of Motor and Wellness Sciences, University of Naples “Parthenope”, Via Ammiraglio Ferdinando Acton, 38, 80133 Naples, Italy

**Keywords:** amyotrophic lateral sclerosis (ALS), metabolomics, NMR, HRMS

## Abstract

Amyotrophic lateral sclerosis (ALS) is a multifactorial neurodegenerative pathology of the upper or lower motor neuron. Evaluation of ALS progression is based on clinical outcomes considering the impairment of body sites. ALS has been extensively investigated in the pathogenetic mechanisms and the clinical profile; however, no molecular biomarkers are used as diagnostic criteria to establish the ALS pathological staging. Using the source-reconstructed magnetoencephalography (MEG) approach, we demonstrated that global brain hyperconnectivity is associated with *early* and *advanced* clinical ALS stages. Using nuclear magnetic resonance (^1^H-NMR) and high resolution mass spectrometry (HRMS) spectroscopy, here we studied the metabolomic profile of ALS patients’ sera characterized by different stages of disease progression—namely *early* and *advanced*. Multivariate statistical analysis of the data integrated with the network analysis indicates that metabolites related to energy deficit, abnormal concentrations of neurotoxic metabolites and metabolites related to neurotransmitter production are pathognomonic of ALS in the *advanced* stage. Furthermore, analysis of the lipidomic profile indicates that *advanced* ALS patients report significant alteration of phosphocholine (PCs), lysophosphatidylcholine (LPCs), and sphingomyelin (SMs) metabolism, consistent with the exigency of lipid remodeling to repair *advanced* neuronal degeneration and inflammation.

## 1. Introduction

Amyotrophic lateral sclerosis (ALS) is a clinically heterogeneous, devastating neurodegenerative disease characterized by muscle wasting, weakness, swallowing impairment, and respiratory failure [1,2]. The incidence and prevalence of ALS are greater in men than in women [3]. The most common pathological phenotype is the spinal one with the onset of limbs and symptoms related to focal muscle weakness and atrophy, mainly in the lower and upper limbs [4].

Numerous scientific evidence claims that ALS pathogenesis is multifactorial [1] and related to different dysmetabolic conditions: glutamate toxicity, oxidative stress, aberrant protein aggregation, defective axonal transport, mitochondrial dysfunction, and altered RNA metabolism.

Evaluation of ALS progression is based on clinical outcomes considering cognitive assessment and the impairment of body sites [1,2]. The most accredited system used for patient clinical evaluation is the King system. It excludes the evaluation of cognitive assessment but considers the clinical stages: stage 1 = impairment of a body site; stage 2 = impairment of two body sites; stage 3 = impairment of 3 body sites; stage 4 = non-invasive ventilation or percutaneous endoscopic gastrostomy [5,6]. King’s staging is performed concurrently with Edinburgh Cognitive and Behavioral ALS Screen (ECAS) [7] and the Amyotrophic Lateral Sclerosis Functional Rating Scale (ALSFRS) [8]. ECAS enables evaluating the patient’s cognitive performance by exploring different domains. The total ECAS score ranges from 0 (worst performance) to 136 (best performance) [9]. Moreover, ALSFR evaluates the functional status of patients. The score ranges from 0 (maximum disability) to 48 (normal condition) [10].

ALS has been extensively investigated in the pathogenetic mechanisms and the clinical profile; however, no molecular biomarkers are used as diagnostic criteria to establish the ALS pathological staging [1,11].

To add new insight into the elucidation of ALS pathogenetic mechanisms, we previously used the source-reconstructed magnetoencephalography (MEG) approach, thus assessing functional brain connectivity in ALS patients compared to sex- and age-matched healthy controls. As a result, we demonstrated that ALS induces a global brain hyperconnectivity, resulting in a less flexible and more vulnerable network. These features vary according to disease severity. *Early* and *advanced* ALS stages differ for a widespread topological reorganization of the brain toward a more integrated and vulnerable network [12,13].

A great deal of evidence has been showing that ALS patients report altered circulating cytokine concentrations, thus indicating that an abnormal neuroinflammatory cascade is associated with ALS [14]. In the hypothesis that the inflammatory processes may have a linear correlation with ALS progression, similarly to the topological alterations on MEG data, we estimated blood levels of a subset interleukin (IL)-4, IL-1β, and interferon-gamma (IFN-γ) of cytokines and compared them with the topological properties of the brain networks. Our data indicated that although circulating cytokine concentrations are significantly different between ALS patients and healthy controls, these differences did not correlate with the topological changes in the functional brain network [15].

Metabolomics provides qualitative and quantitative information on the metabolites present in biological fluids. Therefore, one of the metabolomic applications is the definition of the signatures featuring a specific disease condition.

NMR spectroscopy combined with HRMS represents robust and suitable techniques for metabolomic analysis [16,17,18]. 

Several NMR and HRMS metabolomics studies on cerebrospinal fluid (CSF) and sera have been performed in ALS patients, identifying alterations in biochemical pathways associated with energy expenditure and oxidative stress [19,20,21]. In particular, increased lactic acid and decreased glucose concentration have been found [19,22,23,24,25] to be associated with an excitotoxic role of glutamate [23]. 

Although these metabolomic studies reveal the metabolomic profiles distinguishing ALS patients from healthy controls, a lack of information exists concerning the specificities related to the different stages of ALS disease. These data, in turn, may be clinically relevant to planning appropriate diagnostic and therapeutic interventions [11].

In this context, using ^1^H-NMR spectroscopy and HRMS spectrometry metabolomics, we extended the previously mentioned investigation of ALS patients by analyzing the sera of ALS patients characterized by different stages of disease progression—namely *early* and *advanced*. Multivariate statistical analysis (MVA) of the data integrated with the network analysis confirmed a dysmetabolism in energy pathways. Metabolites related to an energy deficit, abnormal concentrations of neurotoxic metabolites, and metabolites related to neurotransmitter production are pathognomonic in *advanced* ALS patients. Furthermore, analysis of the lipidomic profile indicated that *advanced* ALS patients report significant alteration of phosphocholine (PCs), lysophosphatidylcholine (LPCs), and sphingomyelin (SMs) metabolism, consistent with the exigency of lipid remodeling to repair *advanced* neuronal degeneration and inflammation [12,15]. 

## 2. Materials and Methods

### 2.1. Participants 

Twenty-two ALS patients (15 males, 7 females) were initially recruited from the ALS Center of the First Division of Neurology of the University of Campania “Luigi Vanvitelli” (Naples, Italy). Patients were righthanded and native Italian speakers diagnosed with ALS, according to the revised El-Escorial criteria of ALS. None of the patients showed any mutation in the screened genes SOD1, TARDBP, FUS/TLS, and C9ORF72.

During the recruitment, an anamnestic questionnaire was administered to patients. Participants were asked to specify their eating habits (e.g., Mediterranean, vegetarian or vegan diet) and to report particular lifestyle-related behaviors (e.g., abuse of alcohol or coffee, constant use of psychotropic substances). Subjects with particular habits that could affect the data were excluded from the study.

Out of a total of 22 ALS patients, only 15 (10 males, 5 females) were eligible for recruitment. The remaining 7 were excluded because of (1) unsuitability of blood samples (1 patient); (2) unwillingness to be recruited into a study protocol which, together with the metabolomic profile assessment, involved a more complex panel of clinical evaluations for both diagnostic and non-diagnostic purposes (3 patients); (3) abuse of alcohol (2 patients); (4) constant use of psychotropic drugs (1 patient).

Clinical details and descriptive information about the cohort are shown in Table 1. The study protocol was approved by the local ethics committee, and all participants provided written informed consent in accordance with the Declaration of Helsinki.

### 2.2. Sample Collection and Preparation

Serum samples were collected from males and females according to the standard operating procedure (SOP) for metabolomic-grade serum samples [26]. Serum samples were prepared as previously reported [27,28,29]. Serum was collected and stored in coagulation tubes for serum, centrifuged at 2000 rpm for 10 min, and transferred to 500 µL vials. Before being transferred to a 5 mm heavy-walled NMR tube, samples were spun at 12,000 g using a pre-washed Amicon Ultra-0.5 3000 MWCO filter at 4 °C, to remove proteic and particulate matter [28,30,31]. To prepare NMR samples, 425 μL of each sample was added to 25 μL of 1 M potassium phosphate buffer (pH 7.4) and 50 μL of D_2_O with 0.1% trimethylsilyl propionic-2,2,3,3-d_4_ acid, sodium salt (TSP-d_4_).

### 2.3. NMR Spectroscopy and Processing

NMR experiments were carried out on a Bruker DRX600 spectrometer equipped with a 5 mm triple resonance z-gradient CryoProbe. For spectrometer control and data processing we used TOPSPIN 2.1. Samples were filtered, so 1D-NOESY experiments were acquired at 310 K with the excitation sculpting pulse sequence to suppress the water resonance at a 14 ppm sweep width, 192 transients of 16 k complex points, with an acquisition time of 4 sec transient, and 60 msec mixing time.

^1^H-NMR spectra were processed and analyzed using Bayesil, a software online (http://bayesil.ca (accessed on 12 July 2022)) [30]. Bayesil is a web system that automatically identifies and quantifies metabolites using 1D ^1^H NMR spectra of ultra-filtered biological samples. The attribution of reference chemical shift and quantification was based on the peak intensity of the internal reference compound, TSP-d_4_ (Figure S1, Table S1).

### 2.4. Mass Spectrometry Sample Preparation and Processing

Polar metabolites and lipids were extracted as reported previously [31]. HRMS analyses were performed in flow injection analysis (FIA) by using an Ultimate 3000 UHPLC (Thermo, Bremen, Germany) coupled to a SolariX XR 7T (Bruker Daltonics, Bremen, Germany). The flow rate was set to 10 mL/min and increased in the washing step to 300 mL/min. The instrument was tuned with a standard solution of sodium trifluoracetate (NaTFA). Mass spectra were recorded in a broadband mode in the range of 150–1500 *m/z* for lipids, whereas 90–800 *m/z* was used for polar metabolites, with an ion accumulation of 10 ms. A total of 64 scans were acquired using 4 million data points (4 M), with an approximate resolution of 400.000 at *m/z* 400. Drying gas (nitrogen) was set at 2 mL/min, with a drying temperature of 150 °C. Funnel amplitude was set to 90 V (polar metabolites) or 100 V (lipids), transfer was set at 0.6 MHz, and TOF 0.7 s. Both positive and negative ESI ionization was employed in a separate run. Five replicates of each injection were carried out. The instrument was controlled by Bruker FTMS Control (Bruker). FIA-FT-ICR data extraction, alignment, filtering, and annotation were performed with Metaboscape (v. 5.0, Bruker), as reported previously [31].

### 2.5. Statistical Analysis 

Data matrices were analyzed using the MetaboAnalyst 5.0 by multivariate methods, using PCA (principal component analysis), PLS-DA (partial least-squares discriminant analysis), and O-PLS-DA (orthogonal partial least-squares discriminant analysis). 

Metaboanalyst 5.0 is a comprehensive platform for metabolomics analysis that allows uni- and multivariate statistical analyses on omics datasets. MetaboAnalyst is part of a suite of metabolomic databases, including the human metabolome database (HMDB) [32]. The link to this database allowed us to interpret our data in comparison with those deposited and detectable in healthy subjects’ human biofluids [33,34,35].

Data were normalized using Sum, Log transformed, and Pareto scaled [36]. In particular, the ^1^H NMR data matrix included N = 40 metabolite concentrations; HRMS data matrices included N_polar_ = 49 and N_apolar_ = 98 metabolite concentrations. 

The unsupervised PCA method was carried out to exclude the presence of outliers [37] (Figure S4). Then, supervised PLS-DA and O-PLS-DA analyses were applied to the whole matrix divided into *early* and *advanced* ALS sub-matrices (Table S1). 

Each model was validated using Q2Y and R2Y [38] parameters based on leave-one-out cross validation (LOOCV) and 10-fold cross validation (10-FC) (Tables S2 and S3) to obtain the predictive performance (R package roopls) [39]. Further validation was based on the distance matrix approach carried out by R package MixOmics [40] (Figure S3).

To test the robustness of the discriminant analysis, supervised PLSDA and O-PLS-DA were repeated on two sub-matrices randomly defined. 

Metabolites discriminant for *early* and *advanced* classification were identified using the VIP score analysis (VIP score > 1) on the O-PLS-DA model [39]. The metabolites characterized by VIP score > 1 were further validated as good classifiers by calculating the ROC (receiver operating characteristics) curve (500 bootstrap cycles methods) and AUC (area under the curve) (Tables S4 and S5; Figures S5 and S7) [41,42]. AUC > 70 and *p*-value < 0.05 were considered significant. 

Pathway analysis was performed using MetaboAnalyst 5.0 and Reactome [43,44,45]. 

The pathways corresponding to abnormal metabolite concentrations were identified using the KEGG database based on the number of metabolites involved (hits > 1 and *p*-values < 0.05) [44]. 

The normalized data matrices using Sum, Log transformed, and Pareto scaled was used for the debiased sparse partial correlation (DSPC). The algorithm is based on the de-sparsified graphical lasso modeling procedure and is applied to discover connectivities between a high number of metabolites using fewer samples [46]. DSPC reconstructs a graphical model, where each pair of metabolic characteristics in the dataset is validated from partial correlation coefficients and *p*-values. In the DSPC network, nodes are input metabolites, while the edges represent the connections [46]. 

The network degree and betweenness parameters are calculated for each node. The degree is the number of correlations that a given metabolite establishes with the other metabolites in the network [33,46,47,48]. The betweenness measures the importance of a metabolite for the connections between all pairs of nodes. Metabolites with betweenness values > 35 and node degrees > 5 were considered significant in discriminating the metabolomic profile of the *early* and *advanced* patients; their abnormal concentration affects the concentrations of other metabolites in the network [33,46,48].

The correlation of the ECAS and ALSFR with metabolomic data was evaluated by calculating the Pearson distance [49]. The statistical validation of the correlation was carried out by T-test and false discovery rate (FDR). *p*-value < 0.05, correlation index ≥ ±70 and FDR < 1 were considered significant (Tables S6 and S7).

## 3. Results

### 3.1. Univariate and Multivariate Data Analysis 

The matrix, including metabolites concentrations (N=40) derived from ^1^H NMR spectra, was analyzed using multivariate statistical analysis (Table S1). First, the presence of outliers was ruled out on the whole matrix using the unsupervised PCA method (Figure S4) [37,50]. Then we proceeded to the PLS-DA and O-PLS-DA supervised methods. The O-PLS-DA method uses the individual component as a predictor for the class by improving the discriminatory power of the clusters compared to PLS-DA [51]. 

Aware that the low number of samples can be a limitation for the significance of our analysis, PLS-DA and O-PLS-DA were applied by considering first *early* and *advanced* sub-matrices and then, as an experimental control, two sub-matrices randomly defined. Finally, validation tests were carried out through LOOCV, 10-FC (Tables S2 and S3), and the distance matrix approach [39] (Figure S3). 

**Figure 1:**
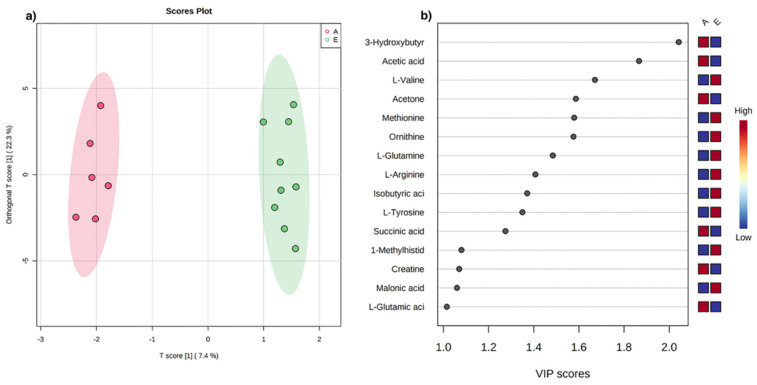
(**a**) O-PLS-DA score plot for ^1^H NMR data collected in 1D-NOESY spectra acquired at 600 MHz. Data represent the sera from 9 *early* (green) and 6 *advanced* (red) ALS patients. (**b**) VIP score analysis derived from O-PLS-DA. Color code indicates higher (red) or lower (blue) concentrations in *early* (E) compared to *advanced* (A) ALS patients.

The score plot in Figure 1a shows that *early* and *advanced* ALS patients define two distinct clusters, each corresponding to a specific metabolomic profile. Q2 = 0.33 by LOOCV and Q2 = 0.30 by 10-fold validate the separation and the distance matrix approach (Figure S3). On the contrary, negative Q2 cross-validation parameters indicated that the dataset for the sub-matrices randomly defined results in no cluster definition (Figure S2). 

The metabolites responsible for cluster separation are identified according to the VIP score analysis (Figure 1b). In particular, the sera of ALS patients in the *advanced* stage report high concentrations of 3-hydroxybutyrate, acetic acid, acetone, succinic acid, L-glutamic acid, creatine, and on the contrary, low concentrations of amino acids such as L-valine, methionine, ornithine, L-glutamine, L-arginine, L-tyrosine, and 1-methylhistidine.

ROC curve was calculated to validate the discriminating power of the metabolites responsible for *early* and *advanced* classification. Significant AUC values (>70, *p*-value < 0.05) were observed for ketone body metabolites, such as 3-hydroxybutyrate, acetone, and acetic acid (Table S4, Figure S5–S7).

**Figure 2:**
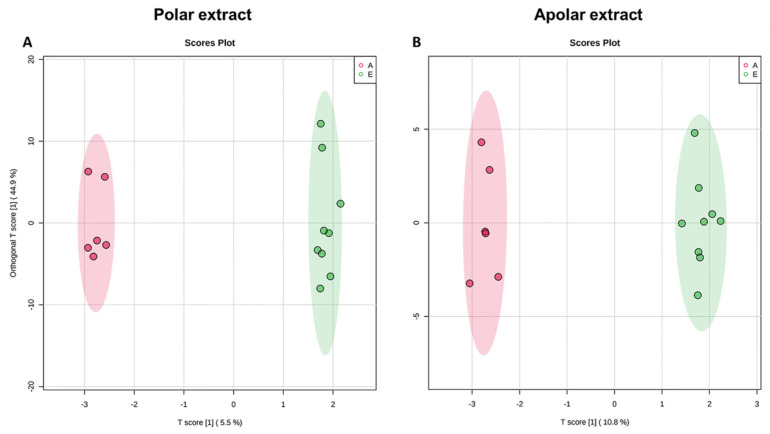
O-PLS-DA score scatter plot for the HRMS data acquired in ESI (+) and (−). Data are relative to polar (**A**) and apolar (**B**) serum extract of 9 *early* (green) and 6 *advanced* (red) ALS patients.

Following the analytical protocol, as previously reported, HRMS analysis of polar and apolar extracts resulted in matrices of metabolite concentrations (N_polar_ = 49, N_apolar_ = 98) for *early* and *advanced* ALS patients (Table S3). The score plot in Figure 2A,B indicates a clear separation of the clusters confirming the existence of different metabolomic profiles. The separation is validated by cross validation (polar extract: Q2 = 0.18 by LOOCV, Q2 = 0.22 by 10-fold; apolar extract: Q2 = 0.59 by LOOCV, Q2 = 0.55 by 10-fold) and distance matrix approach (Figure S3).

**Figure 3:**
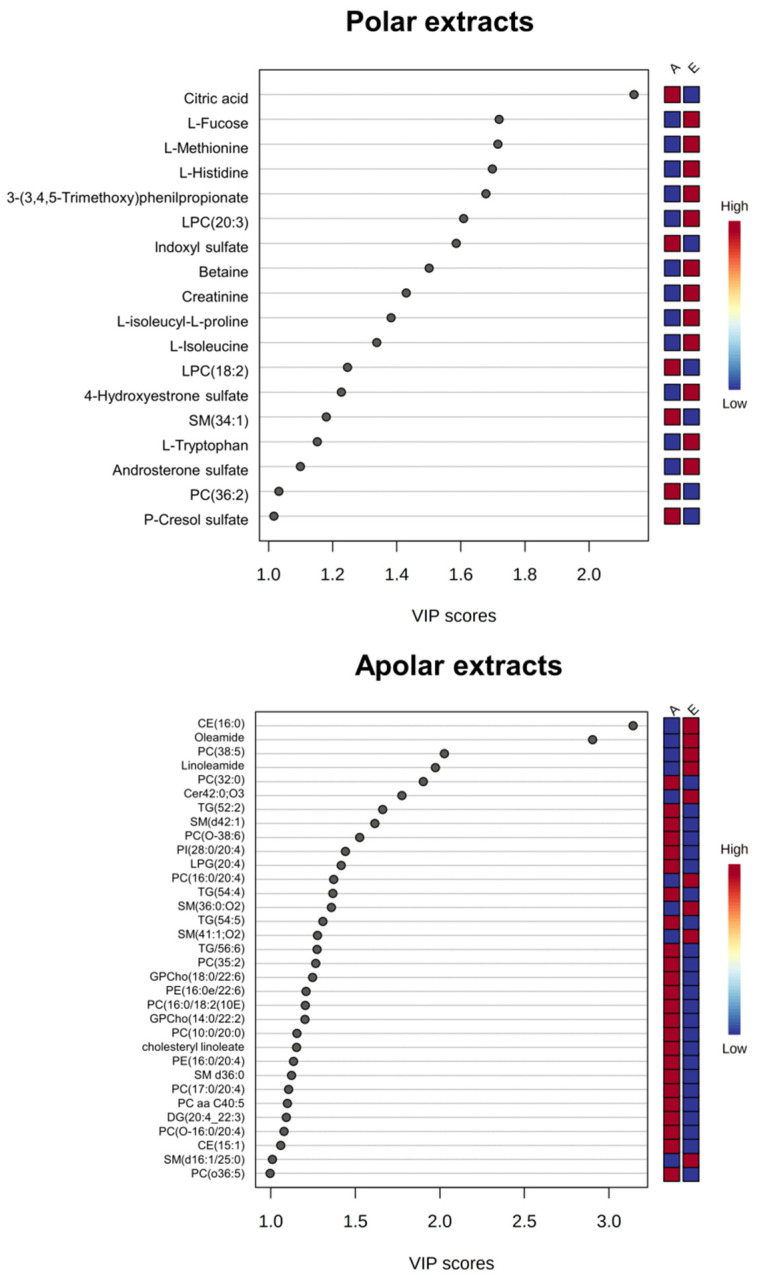
O-PLS-DA VIP graphs for the HRMS data collected using ESI (+) and (−). Data are relative to polar (**top**) and apolar (**bottom**) serum extracts of *early* compared to *advanced* ALS patients. The color code indicates higher (red) or lower (blue) concentrations in *early* (E) compared to *advanced* (A) ALS patients.

VIP score analysis evidenced in *advanced* patients a higher concentration of citric acid and indoxyl sulfate and lower concentrations of some amino acids (methionine, histidine, L-isoleucyl-L-proline, and isoleucine), lysophosphatidylcholine, betaine, and creatinine. Conversely, analysis of the lipid extract showed that in *advanced* ALS patients, there are high concentrations of glycerophospholipids (PC, PI, and PC-Os) and low concentrations of sphingomyelins (SMs) and triacylglycerols (TGs) (Figure 3).

For some of these metabolites — citric acid, L-fucose, 3-(3-4-5-trimethoxyphenyl)propanoic acid, oleic acid, SM 34:1;O2, SM 41:1;O2, PC 36:1 and Cer 42:0;O3 — the discriminant power was confirmed by calculating the ROC curves (AUC > 0.70 *p*-value < 0.05) (Figures S4–S6; Tables S4 and S5).

### 3.2. Debiased Sparse Partial Correlation (DSPC) Algorithm 

**Figure 4:**
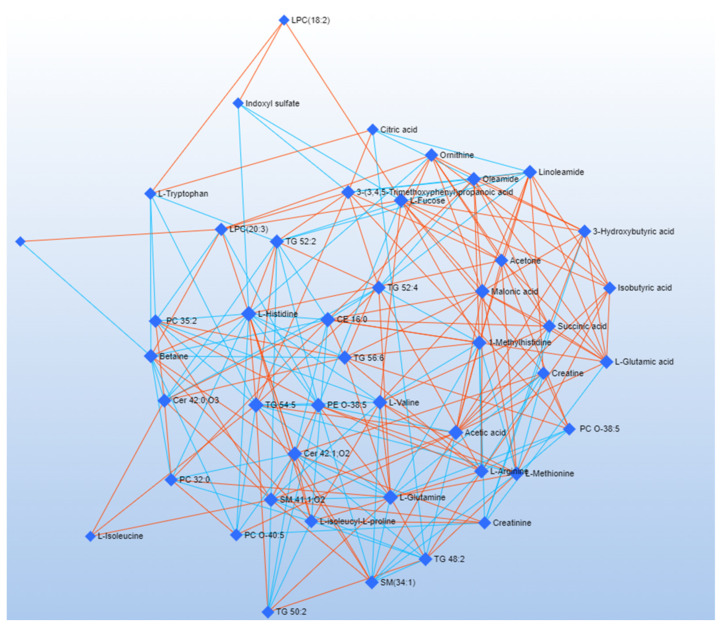
Correlation network obtained by DSPC algorithm using metabolites (VIP > 1) discriminating *early* ALS patients. Nodes represent metabolites. Red lines indicate a direct correlation between metabolites, blue lines indicate an inverse correlation.

**Figure 5:**
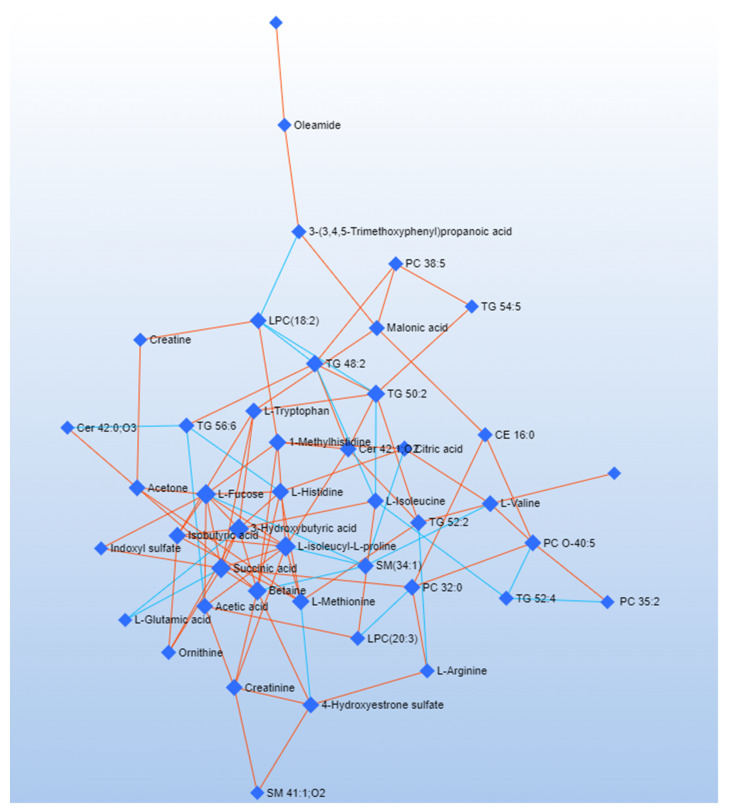
Correlation network obtained by DSPC algorithm using discriminating metabolites (VIP > 1) between *advanced* ALS patients. Nodes represent metabolites; red lines indicate a direct correlation, blue lines indicate an inverse correlation.

DSPC is a machine learning algorithm discovering the connectivities between many metabolites derived from a few samples [52]. Considering that the low number of samples could be a limitation to the significance of our analysis, we used the DSPC to produce an additional validation of our results. DSPC was applied to a single data matrix, including NMR and HRMS data [46]. Figure 4 and Figure 5 show the graphical representation of the DSPC results applied to *early* (Figure 4) and *advanced* (Figure 5) ALS patients. Nodes correspond to the metabolites in input, and the edges represent the correlations among them. Red edges indicate positive correlations, blue edges negative correlations. 

Each node representing the metabolite is characterized by *degree* and *betweenness* values [53] (Table 2). The *degree* refers to the number of connections a node has with other nodes. The *betweenness* represents the number of interconnections. Nodes having a high *degree* and *betweenness* are more likely to be important hubs [54]. We considered a *degree* threshold > 5 and *betweenness* > 35 to identify metabolites distinctive for the pathological phenotype. Network analysis of *advanced* ALS patients indicates that metabolites related to energy pathways (fucose and succinic acid) and ketone bodies (3-hydroxybutyrate) have a central role. Acetic acid and L-histidine are critical in *early* ALS patients, while glycerolipids and betaine have a critical role in both the patient clusters (Table 2).

### 3.3. Combined Pathway Analysis

We carried out pathway analysis to identify the metabolic dysfunctions correlated with *early* and *advanced* ALS pathological stages. To gain meaningful insight from MVA data, we applied metabolic pathway analysis using the whole HRMS and NMR data set. Pathway analysis was performed using MetaboAnalyst 5.0 and Reactome [45,55,56]. Table 3 shows the most significant pathway classified according to hits > 1 and *p*-value < 0.01. The analysis showed the dysregulation of pathways related to lipid metabolisms, such as *Mitochondrial beta-oxidation of Long-Chain Saturated Fatty Acids*, *Beta Oxidation of Very Long Chain Fatty Acids*, and *Oxidation of Branched Chain Fatty Acids metabolism*. In addition, pathway analysis also indicates the dysregulation of neurotransmitter transduction pathways such as *Serotonin receptor and Na^+^/Cl^−^ dependent neurotransmitter transporters* pathway. Interestingly the most discriminant pathway between *early* and *advanced* patients is *Ammonia Recycling,* including *the Urea pathway*, *Glucose-alanine cycle*, and *Glutamate and Glutamine metabolism*.

## 4. Discussion

ALS is a multifactorial neurodegenerative pathology of the upper or lower motor neuron [57]. The causes of the disease include glutamatergic excitotoxicity, oxidative stress, energy deficit, and neuroinflammation [58]. ALS severity is currently assessed by King’s disease staging system based on the number of body site impairments. An accurate definition of the pathological stage supported by multiple biochemical markers is mandatory to develop personalized and successful treatment [57]. Based on our previous MEG study, which evidences specific topological brain networks for *early* and *advanced* ALS patients [12], we performed an exploratory metabolomic study using the blood sera of 9 *early* and 6 *advanced* ALS patients to identify the related metabolomic profile to ALS patients in the different stages of the pathology. 

Concerning the sample size, we know that the number of patients is far less than expected for human study; however, fully conscious of this limitation, we carefully planned our statistical analysis to avoid the misuse and misinterpretation of the data [59]. Accordingly, we used first unsupervised PCA to rule out outliers and then supervised PLS-DA and O-PLS-DA as feature selectors and classifiers. Regarding the application of these methods, since we know they are prone to overfitting, we used several different cross-validation (CV) tests to be confident of the significance of the results. For additional validation, we applied DSPC, a machine learning network analysis suitable for extracting significant information from data matrices containing many variables on a few samples [46]. 

Having said all this, we believe that our study highlights with good confidence a set of metabolites and biochemical pathways that, after additional future validations, may become biomarkers of ALS disease stages. 

Previous scientific studies have shown the correlation between ALS and energetic metabolism abnormality [55,56,60]. Our analyses confirmed a dysmetabolism correlated with energy expenditure. In particular, we observed an increase in ketone bodies in *advanced* ALS patients, suggesting that ALS progression favors glucose deprivation and a metabolic switch to ketone body metabolism [2,61,62]. This metabolic modification is clearly evident in the network analysis, where 3-hydroxybutyric acid reports the highest degree and betweenness score (Table 2). 

Previous evidence identified betaine as a marker of neuroinflammation: betaine plasma concentrations have been found inversely proportional to the severity of motor neuron impairment in ALS [63,64]. Confirming this finding, analysis of serum extracts using HRMS indicated a more pronounced decrease in betaine concentrations in the serum of *advanced* ALS patients [63,64]. The central role of betaine in the pathological metabolic picture of ALS is confirmed by the DSPC analysis, evidencing a critical role of betaine in several networks of both the pathological phenotypes (Table 2).

ALS is known to be associated with neurotoxicity [61,65,66]. Glutamate and indoxyl sulfate, known neurotoxicity markers, are present in abnormally high concentrations in *advanced* ALS patients (Figure 1, Figure 2 and Figure 3). In the same direction, biochemical pathways related to neuroinflammation, such as the Ammonia Recycling pathway (Table 3) (e.g., urea cycle, glutamine, and glutamine metabolism), are more significantly altered in *advanced* ALS patients compared to the *early* ones. 

A low concentration of tyrosine, which is essential for dopamine and epinephrine synthesis [67], is consistent with a general dysmetabolism in the biochemical pathways related to the amine ligand-binding receptors, suggesting a worsening of the neurotransmission deficit depending on the ALS progression [2,68,69] (Table 3). 

Finally, Pearson’s distance correlation analysis showed a direct correlation between tyrosine and the ECAS and ALSFRS-R clinical indices, indicating a relationship between the cognitive and functional improvement of patients and increased tyrosine concentrations (Tables S6 and S7).

Our data point to a significant alteration of the lipid profile in ALS patients [62]. In particular, we observed low circulating serum sphingomyelins (SMs) [70] (Figure 3). These data are coherent with previous ALS and partial lateral sclerosis patients (PLS) follow-up studies, demonstrating a progressive reduction of SMs [71]. According to previous studies on different neurodegenerative diseases, perturbation in the sphingolipid metabolism depends on the necessity of sphingolipid remodeling to increase phospholipid production [72]. Analysis of the lipidome, supported by the DSPC algorithm, reveals an increase in glycerophosphocholine concentrations in *advanced* ALS patients (Figure 3). The alteration of glycerophospholipid levels is associated with neuroinflammation and the dysregulation of cholinergic transmission [73,74]. This is confirmed by the pathways analysis that, in turn, indicates the dysmetabolism of amine ligand-binding receptors and muscarinic acetylcholine receptors (Table 2 and Table 3). 

Considering the male predominance of the pathology, we repeated the MVA on *advanced* and *early* male patients (Figure S8, Table S8). The results confirmed a worsening of energy metabolism, characterized by a shift toward the ketone body metabolism in *advanced* male ALS patients. Furthermore, increased toxicity was evident in male pathological phenotypes as demonstrated by high concentrations of indoxyl-sulfate and glycerophospholipids in *advanced* male patients compared to the *early* ones.

## 5. Conclusions

To identify molecular markers to be used as diagnostic criteria for correct identification of ALS severity, in the present work, we performed a metabolomic study using NMR spectroscopy and HRMS spectrometry on the sera of patients with ALS at *early* and *advanced* stages of disease progression.

MVA on NMR and HRMS data, integrated with network analysis based on machine learning algorithms and supported by biomarkers analysis, indicate that increasing abnormalities in energy expenditure metabolism are typical of ALS patients in the *advanced* disease stage. 

Our multi-omic approach identified the increase in ketone bodies (acetone, 3-hydroxybutyrate, and acetic acid) as pathognomonic of *advanced* ALS stage (Figure 1, Figure 2, Figure 3 and Figure S4, Table 2). Moreover, abnormalities in sphingomyelins concentrations are a datum confirmed by all the approaches, proving that the modification of the lipidomic profile is necessary for lipid remodeling to rebalance neuronal neurodegeneration and inflammation.

## Figures and Tables

**Table 1:** Demographics and clinical information of the participants.

Parameters	ALS “Advanced” Patients Mean (SD) (*n* = 6)	ALS “Early” Patients Mean (SD) (*n* = 9)
Demographic and clinical measures		
Age	64.66 (12.20)	63,92 (10.56)
Male/Female	4/2	6/3
Education	9.17 (4.49)	8.76 (3.67)
Disease duration (months)	54.33 (43.01)	38.68 (16. 22)
ALSFRS-R score	32.0 (8.85)	41.62 (3.60)
UMN score	8.33 (6.77)	5.34 (2.61)
Site of onset	1 bulbar	1 bulbar
	5 Spinal	8 Spinal
Phenotype	2 predominant LMN.	7 predominant LMN.
	1 predominant U.M.N.	1 predominant U.M.N.
	2 Classic	1 Classic
Riluzole	6/6	9/9
Neuropsychological parameters		
ECAS test (total score)	93.67 (14.14)	93.45 (14.82)

ALSFRS-R = Amyotrophic Lateral Sclerosis Functional Rating Scale-Revised; ECAS = Edinburgh Cognitive and Behavioral; LMN = lower motor neuron; UMN = upper motor neuron.

**Table 2:** DSPC network analysis. Degree of node and betweenness of the interrelationships calculated on the metabolites with VIP > 1 related to the *early* and *advanced* ALS patients.

Network Analysis Related to *Early* Patients		
Label	Degree	Betweenness
L-Histidine	16	59.91
TG 54:5	15	58.3
L-Glutamine	15	32.01
Acetic acid	14	45.95
CE 16:0	14	40.1
Cer 42:1;O2	13	33.92
PE O-38:5	13	31.09
L-Valine	13	27.77
Malonic acid	13	23.08
L-Fucose	12	51.26
SM 41:1;O2	12	32.34
1-Methylhistidine	12	24.22
L-Arginine	12	20.99
L-Methionine	12	18.84
Betaine	11	38.48
TG 52:2	11	25.04
TG 56:6	11	24.16
Linoleamide	11	20.78
Oleamide	11	20.36
3-(3,4,5-Trimethoxyphenyl)propanoic acid	10	32.28
Cer 42:0;O3	10	19.77
TG 48:2	10	19.28
L-isoleucyl-L-proline	10	15.43
TG 52:4	10	13.13
L-Glutamic acid	10	10.19
SM(34:1)	10	4.89
Ornithine	9	15.13
Acetone	9	14.81
PC 35:2	9	11.77
3-Hydroxybutyric acid	9	7.81
Succinic acid	9	6.81
LPC(20:3)	8	31.8
Creatinine	8	13.73
Creatine	8	10.52
Isobutyric acid	8	7.92
PC 32:0	8	3.67
PC O-40:5	7	8.09
PC O-38:5	7	6.29
TG 50:2	7	2.66
L-Tryptophan	6	20.66
Citric acid	5	9.23
Indoxyl sulfate	4	6.16
LPC(18:2)	3	3.01
L-Isoleucine	3	0.35
4-Hydroxyestrone sulfate	2	0
**Network analysis related to *advanced* patients**		
**Label**	**Degree**	**Betweenness**
3-Hydroxybutyric acid	10	88.58
L-Isoleucyl-L-proline	10	75.85
L-Fucose	10	70.7
Succinic acid	9	89.14
Betaine	8	74.67
TG 50:2	7	83.66
1-Methylhistidine	6	65.9
TG 48:2	6	64.41
L-Methionine	6	37.13
L-Histidine	6	32.1
Isobutyric acid	6	19.31
LPC(18:2)	5	100.12
L-Valine	5	75.3
L-Isoleucine	5	72.02
PC 32:0	5	65.74
L-Tryptophan	5	62.42
TG 52:2	5	58.58
PC O-40:5	5	54.62
Acetone	5	45.33
SM(34:1)	5	33.5
Acetic acid	5	29.88
4-Hydroxyestrone sulfate	5	29.83
Cer 42:1	5	28.68463
Creatinine	5	27.83
Malonic acid	4	65.18
TG 56:6	4	33.45
Citric acid	4	18.85
3-(3-45-Trimethoxyphenyl-propanoic acid)	3	88.1
CE 16:0	3	28.59
TG 52:4	3	21.78
L-Arginine	3	19.99
LPC(20:3)	3	15.73
PC 38:5	3	11.94
Ornithine	3	0.5
Oleamide	2	42
Creatine	2	9.23

**Table 3:** Metabolic pathway analysis: pathway discriminating *early* and *advanced* ALS patients. For each pathway is reported *p*-value, FDR value.

Pathway	Software	*p*-Value	FDR.
Ammonia Recycling	Metaboanalyst	7.27 × 10^−10^	0.000538
Mitochondrial Beta-Oxidation of Long-Chain Saturated Fatty Acids	Metaboanalyst	0.0125	0.056
Carnitine Synthesis	Metaboanalyst	0.0125	0.0564
Beta Oxidation of Very Long Chain Fatty Acids	Metaboanalyst	0.015	0.0564
Oxidation of Branched Chain Fatty Acids	Metaboanalyst	0.015	0.0564
Amine ligand-binding receptors	Reactome	1.30 × 10^−3^	1.39 × 10^−6^
Serotonin receptors	Reactome	1.10 × 10^−5^	5.89 × 10^−6^
Defective SLC6A19 causes Hartnup disorder (HND)	Reactome	3.80 × 10^−10^	0.008
Na^−^/Cl^−^ dependent neurotransmitter transporters	Reactome	5.13 × 10^−10^	0.009
Cytosolic tRNA aminoacylation	Reactome	8.64 × 10^−9^	0.011
Class A/1 (Rhodopsin-like receptors)	Reactome	9.88 × 10^−10^	0.011
Adrenoceptors	Reactome	1.26 × 10^−12^	0.013
Muscarinic acetylcholine receptors	Reactome	1.47 × 10^−11^	0.014
GPCR ligand binding	Reactome	5.59 × 10^−11^	0.048
Chemokine receptors bind chemokines	Reactome	5.88 × 10^−11^	0.048
Adrenaline signaling through Alpha-2 adrenergic receptor	Reactome	0.001	0.105
Adenylate cyclase inhibitory pathway	Reactome	0.007	0.492

## Data Availability

Data is contained within the article.

## Supplementary Materials

The following supporting information can be downloaded at: https://www.mdpi.com/article/10.3390/metabo12090837/s1, Table S1. Metabolites resulted from serum ALS patients using NMR spectroscopy and HRMS. Table S2. Validation of the multivariate PLS-DA model using LOOCV and 10-Fold related to NMR and HRMS analysis on serum extract of early and advanced ALS patients. Table S3. Validation of the multivariate O-PLS-DA model related to NMR spectroscopy and HRMS analysis on serum extracts of early and advanced ALS patients. Table S4. ROC curve biomarkers related to ALS early and advanced patients’ serum polar extract by NMR analysis. Discriminating metabolites have been classified by AUC (Area under the curve) >70 and *p*-value < 0.05, *p*-value adjustment using Bonferroni. Table S5. ROC curve biomarkers related to ALS early and advanced patients’ serum polar and apolar extract by HRMS analysis. Discriminating metabolites have been classified by AUC (Area under the curve) > 70 and *p*-value < 0.05, *p*-value adjustment using Bonferroni. Table S6. Correlation analysis between the clinical parameter ECAS and ALS patients’ serum metabolites detected by NMR and HRMS. The table shows the correlation coefficient calculated by Person distance and the univariate statistical validation carried out by T-Test, *p*-value, and False discovery rate (FDR). Metabolites with *p*-value < 0.05, correlation index ≥± 70, and FDR<1 are considered to correlate with ECAS parameter. Table S7. Correlation analysis between the clinical parameter ALSFR-S and ALS patients’ serum metabolites detected by NMR and HRMS. The table shows the correlation coefficient calculated by Person distance and the univariate statistical validation carried out by T-Test, *p*-value, and False discovery rate (FDR). Metabolites with *p*-value < 0.05, correlation index ≥±70, and FDR < 1 are considered correlating with ALSFR-S parameter. Table S8. Validation of the multivariate O-PLS-DA model related to NMR spectroscopy and HRMS analysis on serum extracts of early and advanced male ALS patients. Figure S1. 1D-NOESY 1H nuclear magnetic resonance spectra of human sera samples from: early ALS patients (blue) and advanced ALS patient (red). The spectra are acquired at 600 MHz and T = 310 K. Figure S2. PLS-DA score plot for serum polar(A1–B1) and lipid extracts (C1–D1) obtained by mass spectrometry and serum polar extracts obtained by 1H-NMR spectroscopy (E1–F1). The dataset used corresponds to the early (A1–C1–E1) and advanced subset (B1–D1–F1). Histograms (A2–F2) are related to cross-validation indices R2, Q2, and accuracy. Figure S3. Sample prediction area plot carried out using Maximum distance (a,b,c) and Mahalanobis (e,f,g) showing the distribution of samples in validation aerea. Figure S4. PCA and PLS-DA score plot (A–D) for 1H NMR data collected in 1D-NOESY spectra acquired at 600 MHz. Data represent the sera from 9 ALS early patients (green) and 6 ALS advanced patients (red). PCA and PLS-DA score scatter plot for the HRMS data collected acquired in ESI(+) and (−). Data are relative to polar and (B–E) and apolar (C–F) serum extract of 9 ALS early patients (green) compared to 6 advanced patients (red). Figure S5. ROC curve of biomarker identified using polar serum extract by NMR spectroscopy. The sensitivity is on the y-axis, and the specificity is on the x-axis. The AUC is in blue. Figure S6. ROC curve of biomarker identified using serum apolar extract by HRMS spectroscopy. The sensitivity is on the y-axis, and the specificity is on the *x*-axis. The AUC is in blue. Figure S7. ROC curve of biomarker identified on serum polar extract by HRMS spectroscopy. The sensitivity is on the *y*-axis, and the specificity is on the x-axis. The AUC is in blue. Figure S8. OPLS-DA score plot and VIP graph (A–D) for 1H NMR data collected in 1D-NOESY spectra acquired at 600 MHz. Data represent the sera from 6 male ALS early patients (green) and 4 male ALS advanced patients (red). O-PLS-DA score scatter plot and VIP graph for the HRMS data acquired in ESI(+) and (−). Data are relative to polar and (B–E) and apolar (C–F) serum extract of 6 male ALS early patients (green) compared to 4 advanced male patients (red).
