# Supplementary material for: The Metabolomic Profile in Amyotrophic Lateral Sclerosis Changes According to the Progression of the Disease: An Exploratory Study
Source: Metabolites. 2022 Sep 4;12(9):837. doi: 10.3390/metabo12090837 (PMC9504184; doi:10.3390/metabo12090837)
Supplement: Supplementary file 1 [file metabolites-12-00837-s001.zip › metabolites-1839742-supplementary.pdf]

The metabolomic profile in amyotrophic lateral sclerosis changes according to the progression of the disease: a pilot study.

Carmen Marino[1,2,‡], Manuela Grimaldi[2,‡], Eduardo Sommella[2], Tania Ciaglia[2], Angelo Santoro[2], Michela Buonocore[2], Emanuela Salviati[2], Francesca Trojsi[3], Arianna Polverino[4], Pierpaolo Sorrentino[5,6], Giuseppe Sorrentino[4,5,7], Pietro Campiglia[2] and Anna Maria D'Ursi[2,]*

1 Department of Pharmacy and PhD Program in Drug Discovery and Development, University of Salerno, via Giovanni Paolo II, 132, 84084 Fisciano, Salerno, Italy

2 Department of Pharmacy, University of Salerno, via Giovanni Paolo II, 132, 84084 Fisciano, Salerno, Italy

3 Department of Advanced Medical and Surgical Sciences, University of Campania Luigi Vanvitelli. Caserta, Via Maggiore Salvatore Arena, 81100 Contrada San Benedetto, Italy

4 Institute of Diagnosis and Treatment Hermitage Capodimonte, Naples, Cupa delle Tozzole, 2, 80131, Italy.

5 Institute of Applied Sciences and Intelligent Systems of National Research Council, Pozzuoli, Via Campi Flegrei 34, 8007, Italy

6 Department of Engineering, University of Naples "Parthenope", Via Ammiraglio Ferdinando Acton, 38, 80133, Naples, Italy

7 Department of Motor and Wellness Sciences, University of Naples "Parthenope", Via Ammiraglio Ferdinando Acton, 38, 80133, Naples, Italy

* Correspondence: dursi@unisa.it

[†]The first two authors contributed equally.

Table of content:

-Table S1 Metabolites resulted from serum ALS patients using NMR spectroscopy and HRMS.

-Table S2 Validation of the multivariate PLS-DA model using LOOCV and 10-Fold related to NMR and HRMS analysis on serum extract of *early* and a*dvanced* ALS patients.

-Table S3. Validation of the multivariate O-PLS-DA model related to NMR spectroscopy and HRMS analysis on early and advanced ALS patients' serum extracts.

- Table S4. ROC curve biomarkers related to ALS early and *advanced* patients' serum polar extract by NMR analysis. Discriminating metabolites have been classified by AUC (Area under the curve) >70 and p-value< 0.05, p-value adjustment using Bonferroni.

- **Table S5.** ROC curve biomarkers related to ALS *early* and *advanced* patients' serum polar and apolar extract by HRMS analysis. Discriminating metabolites have been classified by AUC>70 and p-value< 0.05, p-value adjustment using Bonferroni.

-**Table S6.** Correlation analysis between the clinical parameter ECAS and ALS patients serum metabolites detected by NMR and HRMS. The table shows the correlation coefficient calculated by Person distance and the univariate statistical validation carried out by T-Test, p-value, and False discovery rate (FDR). Metabolites with p-value <0.05, correlation index ≥±70, and FDR<1 are considered to correlate with ECAS parameter.

Table S7. Correlation analysis between the clinical parameter ALSFR-S and ALS patients' serum metabolites detected by NMR and HRMS. The table shows the correlation coefficient calculated by Person distance and the univariate statistical validation carried out by T-Test, p-value, and False discovery rate (FDR). Metabolites with p-value <0.05, correlation index ≥±70, and FDR<1 are considered correlating with ALSFR-S parameter.

-**Table S8.** Validation of the multivariate O-PLS-DA model related to NMR spectroscopy and HRMS analysis on serum extracts of *early* and *advanced* male ALS patients.

- **Figure S1.** 1D-NOESY ^{1}H nuclear magnetic resonance spectra of human sera samples from: early ALS patients (blue) and advanced ALS patient (red). The spectra are acquired at 600 MHz and T = 310 K.

- **Figure S2.** PLS-DA score plot for serum polar(A1-B1) and lipid extracts (C1-D1) obtained by mass spectrometry and serum polar extracts obtained by ^{1}H-NMR spectroscopy (E1-F1). The dataset used corresponds to the *early* (A1-C1-E1) and *advanced* subset (B1-D1-F1). Histograms (A2-F2) are related to cross-validation indices R2, Q2, and accuracy.

- **Figure S3.** Sample prediction area plot carried out using Maximum distance (a,b,c) and Mahalanobis (e,f,g) showing the distribution of samples in validation aerea.

-**Figure S4**. PCA and PLS-DA score plot (A-D) for ^{1}H NMR data collected in 1D-NOESY spectra acquired at 600 MHz. Data represent the sera from 9 ALS *early* patients (green) and 6 ALS *advanced* patients (red). PCA and PLS-DA score scatter plot for the HRMS data collected acquired in ESI(+) and (-). Data are relative to polar and (B-E) and apolar (C-F) serum extract of 9 ALS *early* patients (green) compared to 6 *advanced* patients (red).

-**Figure S5.** ROC curve of biomarker identified using polar serum extract by NMR spectroscopy. The sensitivity is on the y-axis, and the specificity is on the x-axis. The AUC is in blue.

- **Figure S6.** ROC curve of biomarker identified using serum apolar extract by HRMS spectroscopy. The sensitivity is on the y-axis, and the specificity is on the x-axis. The AUC is in blue.

- **Figure S7.** ROC curve of biomarker identified on serum polar extract by HRMS spectroscopy. The sensitivity is on the y-axis, and the specificity is on the x-axis. The AUC is in blue.

-**Figure S8**. OPLS-DA score plot and VIP graph (A-D) for ^{1}H NMR data collected in 1D-NOESY spectra acquired at 600 MHz. Data represent the sera from 6 male ALS *early* patients (green) and 4 male ALS *advanced* patients (red). O-PLS-DA score scatter plot and VIP graph for the HRMS

data acquired in ESI(+) and (-). Data are relative to polar and (B-E) and apolar (C-F) serum extract of 6 male ALS *early* patients (green) compared to 4 *advanced* male patients (red).

Table S1 Metabolites resulted from serum ALS patients using NMR and HRMS spectroscopy analysis.

NMR spectroscopy	HRMS polar extract	HRMS apolar extract	
1-Methylhistidine	3-(3,4,5-Trimethoxyphenyl) propanoic acid	LPC(18:1)/PC(O-18:1)	PC(O-36:2)/PE(P-39:1)
2-Hydroxybutyrate	3-Methyl-2-oxovaleric acid	3-Deoxyvitamin D3	PC(O-36:3)
Acetic acid	4-Hydroxyestrone sulfate	CAR 11:0	PC(O-36:4)
Betaine	5-Acetylamino-6-formylamino-3-methyluracil	CE (19:0)	PC(O-36:5)
Acetoacetate	9-Decenoylcarnitine	CE(16:0)	PC(O-38:4)
L-Carnitine	Androsterone sulfate	CE(16:1)	PC(O-38:5)
Creatine	Betaine	CE(18:1)	PC(O-38:6)
Citric acid	Citric acid	CE(18:2)	PC(O-40:5)
Choline	Creatine	CE(18:3)	PC(O-40:6)
D-Glucose	Creatinine	CE(20:3)	PC(O-44:5)
Glycine	Decanoylcarnitine	CE(20:4)	PC(P-40:6)
Formic acid	Dehydroepiandrosterone sulfate	Cer 42:0;O3	PE(O-38:5)
Methionine	D-Glucose	Cer 42:1;O2	PI(27:1)
Hypoxanthine	L-Fucose	FA 22:1;O2	PI(29:1)
L-Tyrosine	L-Acetylcarnitine	FA 26:5;O2	PI(38:4)
L-Phenylalanine	L-Arginine	LPC(16:1)	SM 32:1;O2/EPC 35:1;O2
L-Alanine	L-Carnitine	LPC(18:2)	SM 34:1;O2/EPC 37:1;O2
L-Proline	Indoxyl sulfate	LPC(20:4)	SM 34:2;O2/EPC 37:2;O2
L-Threonine	L-Glutamine	MG(18:0)	SM 35:1;O2/EPC 38:1;O2
L-Asparagine	L-Histidine	PC(30:0)/PE(33:0)	SM 36:0;O2
L-Isoleucine	Linoleic acid	PC(32:0)/PE(35:0)	SM 36:2;O2/EPC 39:2;O2
L-Histidine	p-Cresol sulfate	PC(32:1)/PE(35:1)	SM 38:1;O2
Lysine	L-isoleucyl-L-proline	PC(32:2)/PE(35:2)	SM 38:2;O2
L-Serine	L-Lysine	PC(33:1)/PE(36:1)	SM 40:1;O2
L-Lactic acid	L-Methionine	PC(34:1)/PE(37:1)	SM 40:2;O2
L-Aspartic acid	L-Octanoylcarnitine	PC(34:2)/PE(37:2)	SM 41:1;O2
Ornithine	LPC(16:0)	PC(35:1)/PE(38:1)	SM 41:2;O2
Pyruvic acid	LPC(16:1)	PC(35:2)/PE(38:2)	SM 42:1;O2
Succinic acid	LPC(18:0)	PC(35:3)/PE(38:3)	SM 42:2;O2
3-Hydroxybutyric acid	LPC(18:1)	PC(35:4)/PE(38:4)	SM 42:3;O2
L-Arginine	LPC(18:2)	PC(36:1)/PE(39:1)	ST 24:2;O4
Creatinine	LPC(20:3)	PC(36:2)/PE(39:2)	TG(50:1)
L-Glutamine	L-Phenylalanine	PC(36:3)/PE(39:3)	TG(48:1)
L-Leucine	L-Proline	PC(36:4)/PE(39:4)	TG(48:2)
Malonic acid	L-Tryptophan	PC(37:4)/PE(40:4)	TG(50:2)
L-Glutamic acid	L-Tyrosine	PC(38:2)/PE(41:2)	TG(50:3)
L-Valine	Oleic acid	PC(38:3)/PE(41:3)	TG(50:4)
L-Tryptophan	Palmitoleic acid	PC(38:4)/PE(41:4)	TG(51:2)
Acetone	Paraxanthine	PC(38:5)/PE(41:5)	TG(52:2)
Isobutyric acid	PC(34:1)	PC(38:6)/PE(41:6)	TG(52:3)
	PC(34:2)	PC(40:4)/PE(43:4)	TG(52:4)
	PC(36:2)	PC(40:5)	TG(52:5)
	PC(36:3)	PC(40:6)/PE(43:6)	TG(54:4)
	L-Isoleucine	PC(O-16:0)/LPE(19:0)	TG(54:5)

	Phenylalanylphenylalanine<br>SM(34:1)<br>Stearoylcarnitine<br>Uric acid	PC(O-18:0)/LPC(18:0)<br>PC(O-32:1)/PE(O-35:1)<br>PC(O-34:1)/PE(O-37:1)<br>PC(O-34:2)/PE(O-37:2)	TG(56:6)<br>TG(63:4)<br>TG(63:7)<br>TG(66:10)<br>9Z,12Z-octadecadienamide

Table S2 Validation of the multivariate PLS-DA model using LOOCV and 10-Fold-CV related to NMR and HRMS analysis on serum extract of *early* and a*dvanced* ALS patients.

PLS-DA cross-validation related to polar extract using NMR spectroscopy

**Measure using LOOCV**	**1 comps**	**2 comps**	**3 comps**	**4 comps**	**5 comps**
Accuracy	0.67	0.87	1.0	1.0	1.0
R2	0.58	0.85	0.94	0.97	0.98
Q2	0.33	0.61	0.81	0.92	0.95
**Measure using 10 Fold-CV**	**1 comps**	**2 comps**	**3 comps**	**4 comps**	**5 comps**
Accuracy	0.71	0.89	1.0	1.0	1.0
R2	0.59	0.85	0.94	0.97	0.98
Q2	0.30	0.59	0.78	0.87	0.88

PLS-DA cross-validation related to polar extract using HRMS

**Measure using LOOCV**	**1 comps**	**2 comps**	**3 comps**	**4 comps**	**5 comps**
Accuracy	0.75	0.86	1.0	1.0	1.0
R2	0.36	0.73	0.91	0.98	0.99
Q2	0.18	0.53	0.82	0.94	0.98
**Measure using 10 Fold-CV**	**1 comps**	**2 comps**	**3 comps**	**4 comps**	**5 comps**
Accuracy	0.79	0.88	1.0	1.0	1.0
R2	0.36	0.73	0.91	0.98	0.99
Q2	0.22	0.60	0.85	0.95	0.98

PLS-DA cross-validation related to apolar extract using HRMS

Measure using LOOCV	1 comps	2 comps	3 comps	4 comps	5 comps
Accuracy	0.86	1.0	1.0	1.0	1.0
R2	0.82	0.93	0.97	0.99	0.99
Q2	0.59	0.81	0.91	0.97	0.99
**Measure using 10 Fold-CV**	**1 comps**	**2 comps**	**3 comps**	**4 comps**	**5 comps**
Accuracy	0.88	1.0	1.0	1.0	1.0
R2	0.82	0.93	0.97	0.99	0.99
Q2	0.55	0.84	0.93	0.97	0.99

Table S3. Validation of the multivariate O-PLS-DA model related to NMR spectroscopy and HRMS analysis on *early* and *advanced* ALS patient's serum extracts.

**NMR SPECTROSCOPY**				
	p1	o1	o2	o3
R2X	0.0736	0.223	0.121	0.119
R2Y	0.685	0.18	0.0724	0.0241
Q2	0.501	0.216	0.145	0.0593
**HRMS SPECTROSCOPY**				
Polar serum extract				
	p1	o1	o2	o3
R2X	0.108	0.207	0.166	0.088
R2Y	0.855	0.0843	0.0412	0.0109
Q2	0.646	0.25	0.0686	0.0151
Apolar serum extract				
	p1	o1	o2	o3
R2X	0.061	0.238	0.181	0.0861
R2Y	0.56	0.342	0.0568	0.0253
Q2	0.295	0.449	0.144	0.0635

Table S4. ROC curve biomarkers related to ALS early and *advanced* patients' serum polar extract by NMR analysis. Discriminating metabolites have been classified by AUC >70 and p-value< 0.05, p-value adjustment using Bonferroni.

NMR polar extract	AUC	p-value	Bonferroni	ADVANCED	EARLY
Acetic acid	0.85	0.0061	0.000153	↑	↓
3-Hydroxybutyric acid	0.83	0.00082	2.05E-05	↑	↓
Acetone	0.77	0.016	0.0004	↑	↓
L-Glutamine	0.76	0.029	0.000725	↓	↑

Table S5. ROC curve biomarkers related to ALS *early* and *advanced* patients' serum polar and apolar extract by HRMS analysis. Discriminating metabolites have been classified by AUC >70 and p-value< 0.05, p-value adjustment using Bonferroni.

**HRMS polar extract**	**AUC**	**p-value**	**Bonferroni**	**ADVANCED**	**EARLY**
Citric acid	0.94	0.0000000169	1.74227E-10	↑	↓
L-Fucose	0.83	0.0015	1.54639E-05	↓	↑
3-(3-45-Trimethoxyphenyl)propanoic acid	0.80	0.0041	4.2268E-05	↓	↑
Oleic acid	0.80	0.012	0.000123711	↑	↓
SM(34:1)	0.80	0.0017	1.75258E-05	↑	↓
9-Decenoylcarnitine	0.77	0.0067	6.90722E-05	↑	↓
PC(34:2)	0.77	0.03	0.000309278	↑	↓
PC(36:2)	0.77	0.018	0.000185567	↑	↓
LPC(18:2)	0.76	0.0058	5.97938E-05	↑	↓
**HRMS apolar extract**	**AUC**	**p-value**	**Bonferroni**	**ADVANCED**	**EARLY**
SM 41:1;O2	0.86	1.30E-08	2.70833E-10	↓	↑
PC 36:1	0.85	0.00019	3.95833E-06	↓	↑
Cer 42:0;O3	0.80	9.29E-09	1.93542E-10	↓	↑
Oleamide	0.79	4.20E-09	8.75E-11	↓	↑
SM 40:1;O2	0.75	0.015	0.0003125	↓	↑
SM 38:1;O2	0.75	0.0011	2.29167E-05	↓	↑
CE 18:1	0.74	0.0027	0.00005625	↑	↓
Linoleamide	0.74	0.00022	4.58333E-06	↓	↑
TG(52:2)	0.73	0.018	0.000375	↓	↑
PC 38:3	0.73	0.0038	7.91667E-05	↑	↓
SM 41:2;O2	0.72	0.0014	2.91667E-05	↓	↑

Table S6. Correlation analysis between the clinical parameter ECAS and ALS patients' serum metabolites detected by NMR and HRMS. The table shows the correlation coefficient (corr.value) calculated by Person distance and the univariate statistical validation carried out using p-value and False discovery rate (FDR). Metabolites with p-value <0.05, correlation index ≥±70, and FDR<1 are considered correlating with ECAS parameter.

ECAS/NMR metabolites correlation	corr.value	p-value	FDR
1-Methylhistidine	0.81553	4,03E-04	5,64E-03
L-Tyrosine	0.79862	1,23E-03	1,29E-02
L-Histidine	-0.70498	1,36E-01	0.00011452
L-Carnitine	0.69078	2,38E-01	0.00016693
L-Glutamine	0.68096	3,45E-01	0.00020685
L-Alanine	0.64858	0.00010606	0.00054681
Acetoacetate	0.64551	0.00011717	0.00054681
Betaine	0.62283	0.0002371	0.00099581
L-Arginine	0.58847	0.00062501	0.0023864
L-Valine	0.57445	0.00090067	0.0031523
L-Asparagine	-0.55287	0.0015326	0.0049514
Lysine	0.54457	0.0018626	0.0055877
L-Phenylalanine	0.53106	0.002532	0.0067928
L-Glutamic acid	0.53008	0.0025877	0.0067928
Citric acid	0.52225	0.0030727	0.0075913
Pyruvic acid	0.49974	0.0049257	0.011493
L-Aspartic acid	0.49027	0.0059522	0.013158
Hypoxanthine	0.4574	0.011043	0.02319
L-Isoleucine	-0.4333	0.016761	0.033523
Glycine	0.42051	0.020677	0.038745
3-Hydroxybutyric acid	-0.41891	0.021218	0.038745
Succinic acid	-0.39702	0.029828	0.051093
Creatinine	0.39573	0.030413	0.051093
Formic acid	0.38844	0.033901	0.054764
L-Threonine	0.35138	0.056904	0.088518
Acetone	0.32942	0.07546	0.11319
D-Glucose	-0.29875	0.10878	0.15755
L-Proline	0.27714	0.13816	0.19342
L-Leucine	0.26769	0.15267	0.20451
Acetic acid	0.26573	0.15582	0.20451
L-Lactic acid	-0.23562	0.21004	0.26732
L-Serine	-0.20559	0.27575	0.34063
Malonic acid	-0.13932	0.46279	0.55535
Isobutyric acid	0.12223	0.51994	0.60659
2-Hydroxybutyrate	-0.11781	0.53525	0.60758
Creatine	0.11122	0.55845	0.61724
Methionine	0.098429	0.60483	0.65135
L-Tryptophan	0.079341	0.67685	0.69646
Choline	0.078557	0.67988	0.69646

Ornithine	0.036914	0.84644	0.84644
**ECAS/HRMS polar-metabolites correlation**	**corr.value**	**p-value**	**FDR**
Creatine	0.50643	0.0042952	0.071586
LPC(20:3)	0.39038	0.032943	0.30325
L-Arginine	0.38939	0.033426	0.30325
Uric acid	-0.37894	0.038918	0.30325
3-Methyl-2-oxovaleric acid	-0.36255	0.04896	0.30325
PC(36:3)	-0.36217	0.049215	0.30325
L-Isoleucine	-0.35435	0.054701	0.30325
5-Acetylamino-6-formylamino-3-methyluracil	0.34474	0.06209	0.30325
L-isoleucyl-L-proline	-0.33918	0.066716	0.30325
Indoxyl sulfate	-0.321	0.083705	0.34877
PC(34:1)	0.30915	0.096445	0.37094
3-(3-4-5-Trimethoxyphenyl)propanoic acid	0.30017	0.10704	0.38228
L-Tryptophan	0.27287	0.14459	0.44715
p-Cresol sulfate	0.26502	0.15696	0.44715
Creatinine	-0.26292	0.1604	0.44715
L-Carnitine	-0.26257	0.16097	0.44715
PC(36:2)	-0.24119	0.19916	0.51908
LPC(16:0)	0.23255	0.21622	0.51908
Dehydroepiandrosterone sulfate	-0.23167	0.21801	0.51908
L-Proline	0.22454	0.2329	0.52931
Decanoylcarnitine	0.20902	0.26765	0.58185
Linoleic acid	-0.19865	0.29263	0.59681
LPC(16:1)	0.19355	0.30544	0.59681
L-Fucose	0.19164	0.31034	0.59681
Oleic acid	-0.17844	0.34545	0.63973
LPC(18:1)	0.16101	0.39534	0.70597
L-Acetylcarnitine	-0.13345	0.48203	0.77038
Paraxanthine	-0.13319	0.4829	0.77038
L-Octanoylcarnitine	0.13194	0.48706	0.77038
L-Phenylalanine	0.13015	0.49304	0.77038
L-Methionine	0.12236	0.51947	0.78249
Palmitoleic acid	-0.11872	0.53209	0.78249
L-Histidine	0.10728	0.57258	0.81797
L-Tyrosine	0.095787	0.6146	0.84315
L-Glutamine	0.087953	0.64396	0.84315
Phenylalanylphenylalanine	-0.087277	0.64652	0.84315
LPC(18:2)	0.084346	0.65766	0.84315
4-Hydroxyestrone sulfate	-0.067635	0.7225	0.90313
SM(34:1)	0.056344	0.76743	0.9359
LPC(18:0)	-0.040053	0.83356	0.93884
Stearoylcarnitine	0.038924	0.83819	0.93884
Androsterone sulfate	-0.037514	0.84398	0.93884
Citric acid	-0.037276	0.84496	0.93884
PC(34:2)	-0.020171	0.91575	0.98612
Betaine	0.0099735	0.95828	0.98612
D-Glucose	-0.0073776	0.96914	0.98612
L-Lysine	-0.0042401	0.98226	0.98612

9-Decenoylcarnitine	-0.0033163	0.98612	0.98612
**ECAS/HRMS apolar-metabolites correlation**	**corr.value**	**p-value**	**FDR**
PI(29:1)	0.44006	0.014952	0.33793
PC(O-32:1)/PE(O-35:1)	0.39702	0.029831	0.33793
PC(O-16:0)/LPE(19:0)	0.39549	0.030525	0.33793
PC(P-40:6)	0.38872	0.03376	0.33793
PC(30:0)/PE(33:0)	0.38446	0.035934	0.33793
PC(35:1)/PE(38:1)	0.38271	0.036859	0.33793
SM 34:1;O2/EPC 37:1;O2	0.37518	0.041058	0.33793
PC(O-18:0)/LPC(18:0)	0.37461	0.04139	0.33793
PC(O-44:5)	0.36716	0.045947	0.33793
TG(48:2)	0.36123	0.04985	0.33793
TG(52:2)	0.35884	0.051491	0.33793
TG(48:1)	0.35703	0.052769	0.33793
PC(38:3)/PE(41:3)	0.35197	0.056466	0.33793
PI(27:1)	0.34464	0.062169	0.33793
PC(38:5)/PE(41:5)	0.34348	0.063115	0.33793
LPC(16:1)	-0.33757	0.0681	0.33793
PC(36:1)/PE(39:1)	0.33517	0.070211	0.33793
TG(50:4)	0.3322	0.072881	0.33793
SM 35:1;O2/EPC 38:1;O2	0.32709	0.07768	0.33793
PE(O-38:5)	0.32701	0.077756	0.33793
TG(54:5)	0.32541	0.079311	0.33793
TG(52:3)	0.31632	0.088568	0.35527
PC(40:5)	0.3144	0.09063	0.35527
TG(50:3)	0.30916	0.096435	0.36349
PC(40:4)/PE(43:4)	0.30555	0.1006	0.36513
PC(O-36:2)/PE(P-39:1)	0.29633	0.11183	0.3914
PC(38:4)/PE(41:4)	0.2918	0.11767	0.39764
ST 24:2;O4	0.28541	0.12631	0.40491
CE(20:4)	0.28308	0.12957	0.40491
PC(O-36:4)	0.28122	0.13221	0.40491
CE (19:0)	-0.2773	0.13792	0.40556
9Z-12Z-octadecadienamide	0.27424	0.14251	0.40556
CE(16:0)	-0.2723	0.14546	0.40556
TG(50:2)	0.27003	0.14898	0.40556
PC(O-38:4)	0.26674	0.1542	0.40841
PC(34:1)/PE(37:1)	0.26022	0.16489	0.42269
PC(O-40:5)	0.25826	0.16821	0.42269
PC(O-36:3)	0.2457	0.19061	0.467
SM 42:3;O2	0.24195	0.1977	0.46988
CE(18:1)	-0.24003	0.20138	0.46988
PC(36:4)/PE(39:4)	0.22672	0.22829	0.52028
TG(52:5)	0.21143	0.26204	0.58364

PC(O-36:5)	0.2088	0.26815	0.58398
SM 42:1;O2	0.2	0.28931	0.60325
PC(35:2)/PE(38:2)	-0.19583	0.29967	0.60867
PC(40:6)/PE(43:6)	0.19327	0.30617	0.60867
SM 40:1;O2	0.19156	0.31055	0.60867
PC(34:2)/PE(37:2)	-0.18455	0.32892	0.62564
TG(56:6)	0.17962	0.34222	0.62564
TG(51:2)	0.17708	0.34921	0.62564
PC(37:4)/PE(40:4)	0.17523	0.35436	0.62564
PC(32:2)/PE(35:2)	-0.17373	0.35855	0.62564
MG(18:0)	-0.17354	0.35908	0.62564
SM 36:0;O2	-0.16916	0.37151	0.62564
PC(32:0)/PE(35:0)	0.16718	0.37723	0.62564
PC(35:4)/PE(38:4)	-0.16095	0.39551	0.62564
PC(36:2)/PE(39:2)	0.15954	0.39971	0.62564
CE(18:3)	-0.15936	0.40027	0.62564
TG(63:7)	-0.15926	0.40056	0.62564
CE(16:1)	0.15753	0.40576	0.62564
LPC(18:2)	0.1566	0.40858	0.62564
PC(38:2)/PE(41:2)	0.15351	0.41799	0.62578
PC(38:6)/PE(41:6)	0.15239	0.42144	0.62578
SM 32:1;O2/EPC 35:1;O2	0.15027	0.42801	0.62604
LPC(20:4)	-0.14388	0.44814	0.64586
TG(66:10)	0.1394	0.46253	0.65692
SM 38:2;O2	0.13242	0.48547	0.67965
Cer 42:0;O3	-0.12943	0.49544	0.68319
CE(18:2)	-0.1275	0.50194	0.68319
PC(33:1)/PE(36:1)	-0.12336	0.51604	0.69277
PC(35:3)/PE(38:3)	-0.11599	0.54162	0.70713
SM 38:1;O2	0.11574	0.5425	0.70713
TG(63:4)	0.11208	0.5554	0.70713
FA 26:5;O2	-0.11127	0.55828	0.70713
TG(54:4)	0.11	0.56282	0.70713
SM 40:2;O2	0.10417	0.58383	0.71889
TG(50:1)	0.10219	0.59104	0.71889
CE(20:3)	0.10133	0.59418	0.71889
PC(O-40:6)	-0.095417	0.61598	0.73617
3-Deoxyvitamin D3	0.092043	0.62856	0.73778
PC(36:3)/PE(39:3)	-0.091024	0.63239	0.73778
PC(O-38:6)	-0.079254	0.67719	0.78076
TG(52:4)	0.073682	0.69879	0.79446
FA 22:1;O2	0.07202	0.70528	0.79446
SM 34:2;O2/EPC 37:2;O2	0.062699	0.74204	0.82637
PC(O-38:5)	0.059382	0.75527	0.83164

CAR 11:0	0.055348	0.77144	0.83344
PC(O-34:1)/PE(O-37:1)	0.054734	0.77391	0.83344
PC(32:1)/PE(35:1)	-0.051512	0.7869	0.83822
SM 36:2;O2/EPC 39:2;O2	0.041395	0.82806	0.87258
SM 41:2;O2	0.037244	0.84509	0.88105
SM 42:2;O2	0.028796	0.87994	0.90772
PI(38:4)	0.0059784	0.97499	0.98892
Cer 42:1;O2	-0.0050589	0.97883	0.98892
SM 41:1;O2	-0.0024445	0.98977	0.98977

Table S7. Correlation analysis between the clinical parameter ALSFR-S and ALS patients' serum metabolites detected by NMR and HRMS. The table shows the correlation coefficient (corr.value) calculated by Person distance and the univariate statistical validation carried out using p-value and False discovery rate (FDR). Metabolites with p-value <0.05, correlation index ≥±70 and FDR<1 are considering correlating with ALSFR-S parameter.

ALSFR-S/NMR metabolites correlation	Corr.value	p-value	FDR
L-Tyrosine	0.82867	1,56E-05	2,19E-03
1-Methylhistidine	0.7837	3,03E-03	3,18E-02
L-Histidine	-0.72426	6,05E-02	4,39E-01
L-Glutamine	0.72344	6,27E-02	4,39E-01
L-Alanine	0.7068	1,27E-01	7,60E-01
Betaine	0.69198	2,28E-01	0.00011957
L-Carnitine	0.65582	8,34E-01	0.00038939
Acetoacetate	0.64071	0.00013665	0.00057391
L-Arginine	0.62769	0.00020483	0.00078207
L-Valine	0.57459	0.00089746	0.0031411
L-Glutamic acid	0.56481	0.001147	0.0037058
L-Asparagine	-0.55928	0.0013136	0.0039408
Citric acid	0.53845	0.0021436	0.0058707
Lysine	0.53659	0.0022364	0.0058707
L-Aspartic acid	0.53375	0.0023841	0.0058901
L-Phenylalanine	0.50264	0.004643	0.010834
L-Isoleucine	-0.49251	0.0056949	0.012589
Pyruvic acid	0.48848	0.0061662	0.012949
Hypoxanthine	0.41339	0.023169	0.043549
Glycine	0.41257	0.023469	0.043549
L-Threonine	0.41155	0.023848	0.043549
Succinic acid	-0.35986	0.050786	0.088875
Formic acid	0.35215	0.056328	0.094631

3-Hydroxybutyric acid	-0.33927	0.066637	0.10764
D-Glucose	-0.3272	0.077571	0.12067
Acetone	0.30914	0.096454	0.14468
Creatinine	0.28929	0.12101	0.17526
L-Lactic acid	-0.28134	0.13204	0.18486
L-Proline	0.26255	0.16101	0.21815
Acetic acid	0.24006	0.20133	0.26424
L-Serine	-0.22669	0.22835	0.29062
L-Leucine	0.21991	0.24294	0.3001
Methionine	0.18497	0.3278	0.39335
2-Hydroxybutyrate	-0.15512	0.41309	0.48194
L-Tryptophan	0.13046	0.49199	0.55847
Malonic acid	-0.11984	0.52819	0.58379
Creatine	0.11334	0.55093	0.59331
Ornithine	0.10216	0.59113	0.62069
Isobutyric acid	0.087394	0.64608	0.66183
Choline	0.02829	0.88203	0.88203
**ALSFR-S/HRMS polar-metabolites correlation**	**Corr.value**	**p-value**	**FDR**
PC(34:1)	0.44135	0.014627	0.15476
Uric acid	-0.43182	0.01718	0.15476
Dehydroepiandrosterone sulfate	-0.43073	0.017495	0.15476
L-isoleucyl-L-proline	-0.42711	0.018571	0.15476
LPC(20:3)	0.35765	0.052327	0.33513
Creatinine	-0.35584	0.053621	0.33513
L-Isoleucine	-0.33027	0.074667	0.41482
Creatine	0.27134	0.14694	0.70556
PC(36:3)	-0.25058	0.18169	0.70556
3-(3-45-Trimethoxyphenyl)propanoic acid	0.24108	0.19937	0.70556
L-Methionine	0.23037	0.22067	0.70556
5-Acetylamino-6-formylamino-3-methyluracil	0.22867	0.22419	0.70556
Indoxyl sulfate	-0.22774	0.22614	0.70556
4-Hydroxyestrone sulfate	-0.21907	0.24478	0.70556
SM(34:1)	0.19602	0.29921	0.70556
Palmitoleic acid	-0.19303	0.30678	0.70556
Betaine	-0.19281	0.30734	0.70556
LPC(18:2)	-0.19237	0.30848	0.70556
LPC(18:1)	0.1885	0.3185	0.70556
L-Arginine	0.18528	0.32699	0.70556
L-Tryptophan	0.18461	0.32875	0.70556
Stearoylcarnitine	-0.18093	0.33867	0.70556
L-Histidine	-0.17338	0.35953	0.71906
LPC(16:1)	0.16194	0.39259	0.74606
Oleic acid	-0.15849	0.40287	0.74606
LPC(16:0)	0.14322	0.45024	0.804

L-Phenylalanine	0.11748	0.5364	0.84022
L-Octanoylcarnitine	0.11716	0.5375	0.84022
L-Carnitine	-0.11062	0.5606	0.84022
Decanoylcarnitine	0.10584	0.57777	0.84022
PC(36:2)	-0.10294	0.58828	0.84022
L-Lysine	-0.09893	0.60298	0.84022
Androsterone sulfate	-0.094915	0.61784	0.84022
LPC(18:0)	0.093616	0.62268	0.84022
9-Decenoylcarnitine	-0.089618	0.63768	0.84022
PC(34:2)	-0.089382	0.63857	0.84022
L-Tyrosine	0.079668	0.67559	0.86614
Paraxanthine	0.070872	0.70978	0.87131
L-Glutamine	0.067332	0.7237	0.87131
D-Glucose	-0.064775	0.7338	0.87131
p-Cresol sulfate	0.060394	0.75123	0.87131
Citric acid	-0.056513	0.76676	0.87131
L-Fucose	0.041534	0.82749	0.91944
3-Methyl-2-oxovaleric acid	-0.035412	0.85262	0.92676
L-Proline	0.026918	0.88772	0.94438
L-Acetylcarnitine	0.018708	0.92183	0.95194
Linoleic acid	0.016053	0.9329	0.95194
Phenylalanylphenylalanine	0.0001226	0.99949	0.99949
**ALSFR-S/HRMS apolar-metabolites correlation**	**Corr.value**	**p-value**	**FDR**
TG(50:3)	0.56605	0.0011124	0.024807
ST 24:2;O4	0.55956	0.0013045	0.024807
TG(52:3)	0.5471	0.0017559	0.024807
TG(52:2)	0.54458	0.001862	0.024807
PC(O-16:0)/LPE(19:0)	0.54003	0.0020681	0.024807
SM 35:1;O2/EPC 38:1;O2	0.53498	0.0023193	0.024807
9Z-12Z-octadecadienamide	0.53206	0.002476	0.024807
PC(38:5)/PE(41:5)	0.52257	0.0030511	0.024807
PC(35:1)/PE(38:1)	0.52021	0.0032112	0.024807
PI(29:1)	0.52006	0.0032218	0.024807
TG(48:1)	0.51724	0.0034227	0.024807
TG(52:5)	0.51298	0.0037464	0.024807
PC(O-36:2)/PE(P-39:1)	0.51194	0.003829	0.024807
TG(54:5)	0.50926	0.0040502	0.024807
PC(35:4)/PE(38:4)	-0.47718	0.0076681	0.042709
PC(O-32:1)/PE(O-35:1)	0.47598	0.0078446	0.042709
TG(50:2)	0.45568	0.011387	0.057769
PC(36:1)/PE(39:1)	0.45373	0.01179	0.057769
CE(18:3)	-0.44565	0.013582	0.062573
SM 42:1;O2	0.44371	0.014047	0.062573
PE(O-38:5)	0.43702	0.015745	0.067088

PC(O-18:0)/LPC(18:0)	0.43371	0.016645	0.067968
PC(40:5)	0.41652	0.022043	0.083775
TG(50:4)	0.41601	0.022226	0.083775
PC(40:4)/PE(43:4)	0.41153	0.023857	0.086593
TG(48:2)	0.40736	0.025461	0.089113
TG(52:4)	0.40127	0.027967	0.09024
PI(27:1)	0.40026	0.028399	0.09024
TG(63:4)	0.39993	0.028545	0.09024
LPC(16:1)	-0.39597	0.030306	0.090339
SM 34:1;O2/EPC 37:1;O2	0.39423	0.031106	0.090339
PC(36:2)/PE(39:2)	0.39301	0.031681	0.090339
PC(38:4)/PE(41:4)	0.38978	0.033235	0.090339
PC(37:4)/PE(40:4)	0.38976	0.033246	0.090339
3-Deoxyvitamin D3	0.38653	0.034861	0.090339
PC(34:1)/PE(37:1)	0.3862	0.035029	0.090339
PC(38:3)/PE(41:3)	0.3759	0.040642	0.10026
SM 40:1;O2	0.37457	0.041416	0.10026
PC(O-36:4)	0.37367	0.041946	0.10026
CE(18:2)	-0.36753	0.045711	0.1062
TG(54:4)	0.36013	0.050603	0.11083
CE (19:0)	-0.35971	0.050889	0.11083
PC(35:3)/PE(38:3)	-0.35658	0.053091	0.11311
MG(18:0)	-0.34324	0.063316	0.13202
CAR 11:0	0.32682	0.077935	0.15621
TG(66:10)	0.32513	0.07958	0.15621
PC(35:2)/PE(38:2)	-0.32501	0.079699	0.15621
PC(38:6)/PE(41:6)	0.32189	0.082803	0.15911
PC(O-38:4)	0.31611	0.08879	0.16734
CE(20:3)	0.31304	0.092114	0.17032
PC(36:4)/PE(39:4)	0.31053	0.094887	0.1722
CE(16:0)	-0.30048	0.10666	0.18894
TG(51:2)	0.29941	0.10797	0.18894
PC(O-36:3)	0.29652	0.11158	0.19184
LPC(20:4)	-0.28081	0.13281	0.2244
PC(O-36:5)	0.27919	0.13515	0.22449
TG(56:6)	0.27001	0.14901	0.24339
SM 40:2;O2	0.25032	0.18216	0.29265
SM 41:2;O2	0.23891	0.20357	0.32177
PC(38:2)/PE(41:2)	0.23179	0.21777	0.33875
SM 42:3;O2	0.22875	0.22403	0.34305
Cer 42:1;O2	0.21603	0.25156	0.37928
CE(18:1)	-0.21329	0.25778	0.38276
TG(50:1)	0.20366	0.28038	0.40496
PC(30:0)/PE(33:0)	0.20341	0.28099	0.40496

PC(33:1)/PE(36:1)	-0.19635	0.29838	0.42125
PC(O-44:5)	0.19535	0.30089	0.42125
PC(O-34:1)/PE(O-37:1)	0.18465	0.32864	0.45362
PC(P-40:6)	0.18077	0.33909	0.46154
PC(O-40:5)	0.17581	0.35273	0.47353
SM 41:1;O2	0.16763	0.37593	0.48825
SM 36:0;O2	0.16721	0.37716	0.48825
PI(38:4)	-0.16669	0.37865	0.48825
PC(34:2)/PE(37:2)	-0.16154	0.39375	0.50114
CE(16:1)	0.14887	0.43236	0.53935
PC(O-38:5)	0.1481	0.43479	0.53935
Cer 42:0;O3	0.14462	0.44576	0.54604
SM 36:2;O2/EPC 39:2;O2	0.14288	0.45132	0.54604
PC(32:2)/PE(35:2)	-0.13939	0.46257	0.55106
TG(63:7)	0.13509	0.47661	0.55106
SM 42:2;O2	-0.13473	0.47779	0.55106
PC(O-38:6)	0.13468	0.47796	0.55106
SM 32:1;O2/EPC 35:1;O2	-0.11545	0.54352	0.61936
SM 34:2;O2/EPC 37:2;O2	0.083699	0.66013	0.73575
PC(40:6)/PE(43:6)	0.083557	0.66067	0.73575
PC(32:1)/PE(35:1)	0.071262	0.70825	0.77987
LPC(18:2)	0.063442	0.73909	0.80479
PC(O-40:6)	-0.051093	0.7886	0.84926
FA 22:1;O2	0.024127	0.8993	0.95184
PC(32:0)/PE(35:0)	-0.023169	0.90327	0.95184
SM 38:1;O2	-0.020009	0.91642	0.95541
PC(36:3)/PE(39:3)	0.016959	0.92912	0.95847
CE(20:4)	0.0066675	0.97211	0.98919
SM 38:2;O2	0.0049966	0.97909	0.98919
FA 26:5;O2	0.0010099	0.99577	0.99577

Table S8. Validation of the multivariate O-PLS-DA model related to NMR spectroscopy and HRMS analysis on *early* and *advanced* male ALS patients' serum extracts.

**NMR SPECTROSCOPY**			
	p1	o1	o2
R2X	0.16	0.16	0.15
R2Y	0.83	0.15	0.20
Q2	0.66	0.23	0.14
**HRMS SPECTROSCOPY**			
Polar serum extract			
	p1	o1	o2
R2X	0.19	0.26	0.12

R2Y	0.78	0.17	0.03
Q2	0.69	0.19	0.07
Apolar serum extract			
	p1	o1	o2
R2X	0.05	0.42	0.12
R2Y	0.74	0.15	0.09
Q2	0.10	0.47	0.35

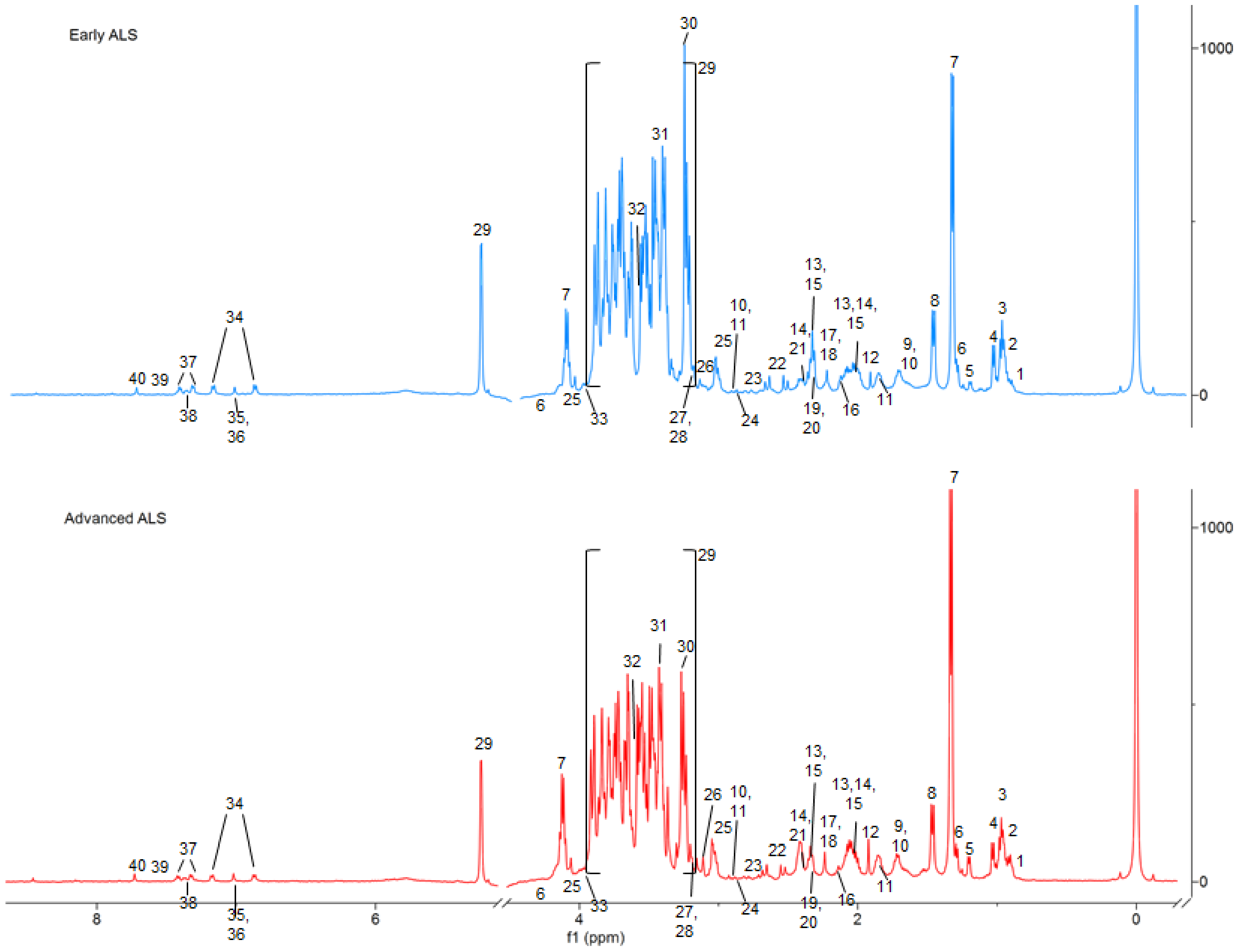

1: 2-Hydroxybutyrate; 2: Isoleucine; 3: Leucine; 4: Valine; 5: 3-Hydroxybutyrate; 6: L-Threonine; 7: Lactate; 8: Alanine; 9: Arginine; 10: Lysine; 11: Ornithine; 12: Acetate; 13: L-Proline; 14: Glutamine; 15: Glutamate; 16: Methionine; 17: Acetone; 18: Acetoacetate; 19: Pyruvate; 20: Isobutyrate; 21: Succinate; 22: Citrate; 23: Aspartate; 24: Asparagine; 25: Creatinine; 26: Malonate; 27: Choline; 28: Carnitine; 29: D-Glucose; 30: Betaine; 31: Glycine; 32: Serine; 33: Creatine; 34: Tyrosine; 35: Histidine; 36: 1-Methylhistidine; 37: Phenylalanine; 38: Tryptophan; 39: Hypoxanthine; 40: Formate.

Figure S1. 1D-NOESY ^{1}H nuclear magnetic resonance spectra of human sera samples from: early ALS patients (blue) and advanced ALS patient (red). The spectra are acquired at 600 MHz and T = 310 K.

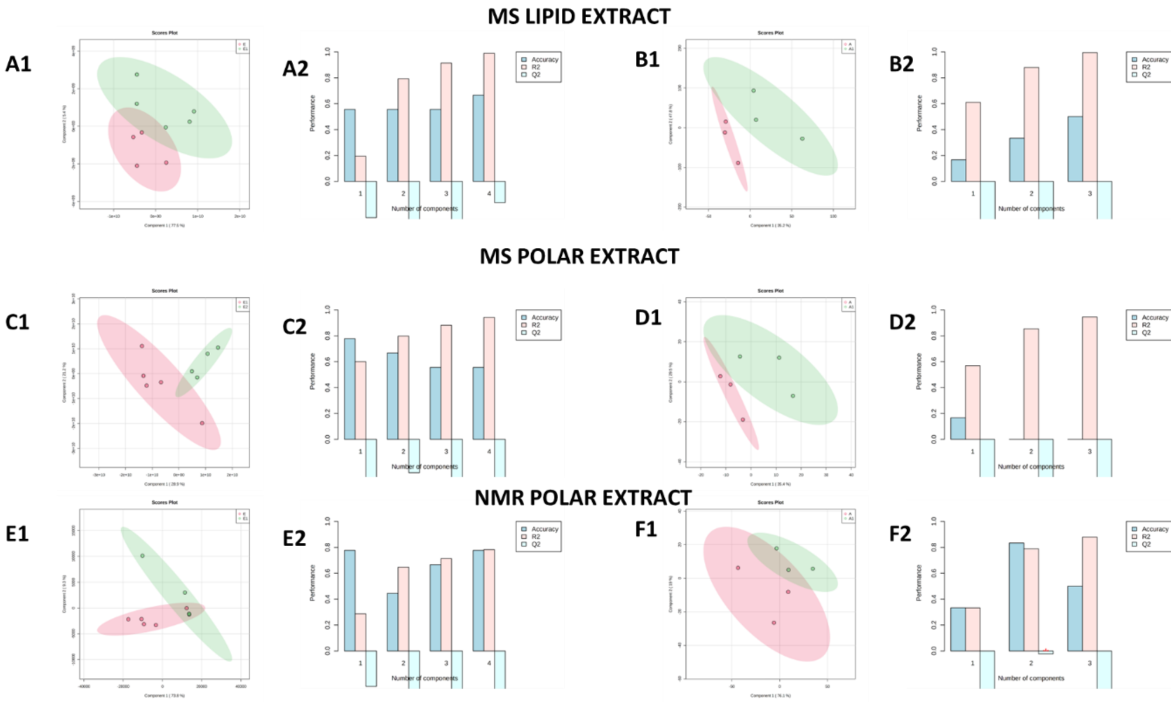

Figure S2. PLS-DA score plot for serum polar(A1-B1) and lipid extracts (C1-D1) obtained by mass spectrometry and serum polar extracts obtained by ^{1}H-NMR spectroscopy (E1-F1). The dataset used corresponds to *early* (A1-C1-E1) and *advanced* subset (B1-D1-F1). Histograms (A2-F2) are related to cross-validation indices R2, Q2 and accuracy.

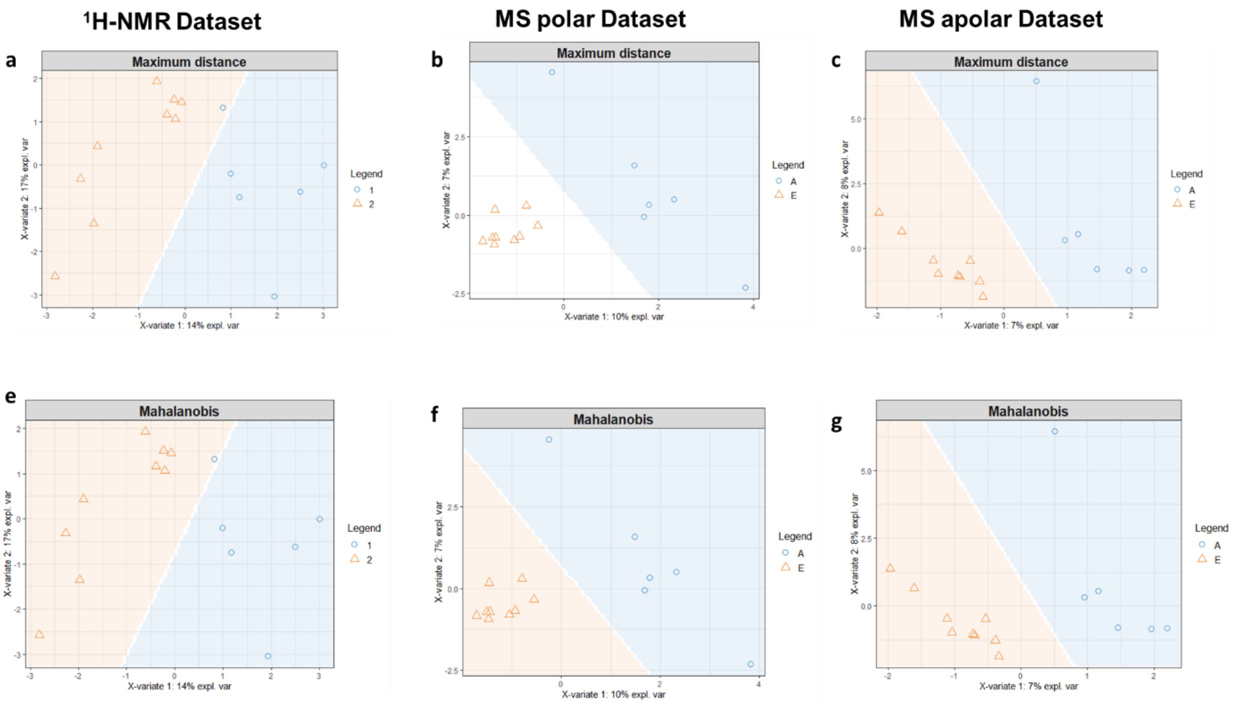

Figure S3. Sample prediction area plot carried out using Maximum distance (a,b,c) and Mahalanobis (e,f,g) showing the distribution of samples in validation aerea.

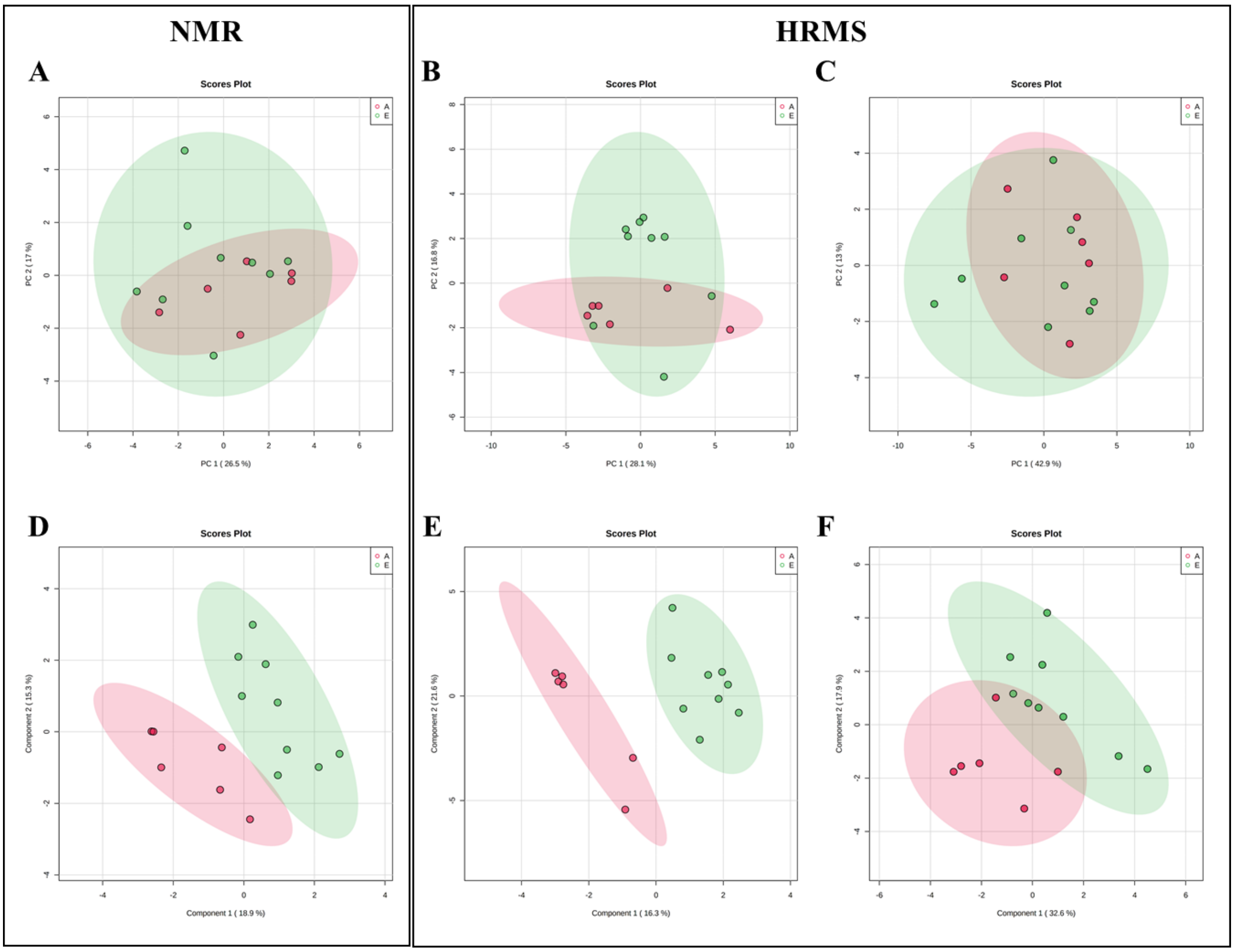

Figure S4. PCA and PLS-DA score plot (A-D) for ^{1}H NMR data collected in 1D-NOESY spectra acquired at 600 MHz. Data represent the sera from 9 ALS *early* patients (green) and 6 ALS *advanced* patients (red). PCA and PLS-DA score scatter plot for the HRMS data collected acquired in ESI(+) and (-). Data are relative to polar and (B-E) and apolar (C-F) serum extract of 9 ALS *early* patients (green) compared to 6 *advanced* patients (red).

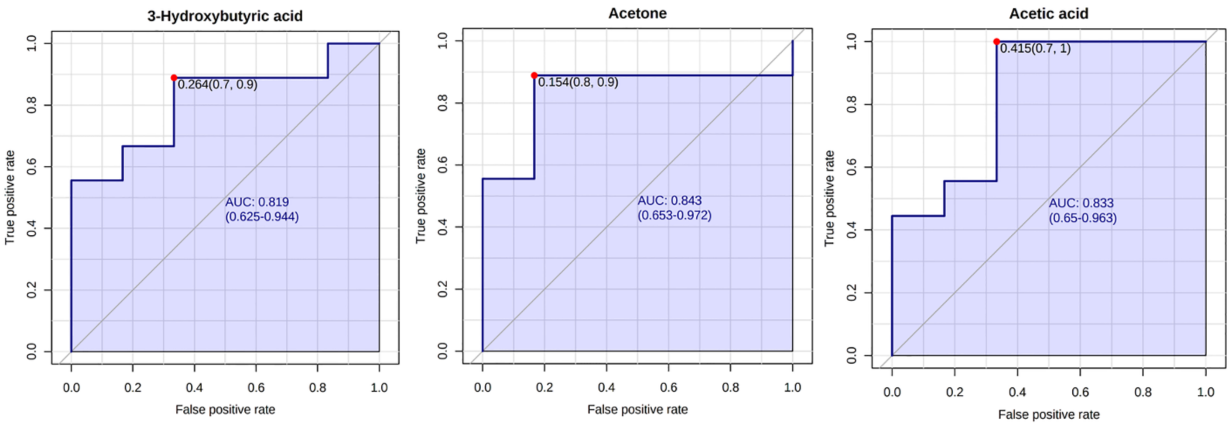

Figure S5. ROC curve of biomarker identified using serum polar extract by NMR spectroscopy. The sensitivity is on the y-axis, and the specificity is on the x-axis. AUC is in blue.

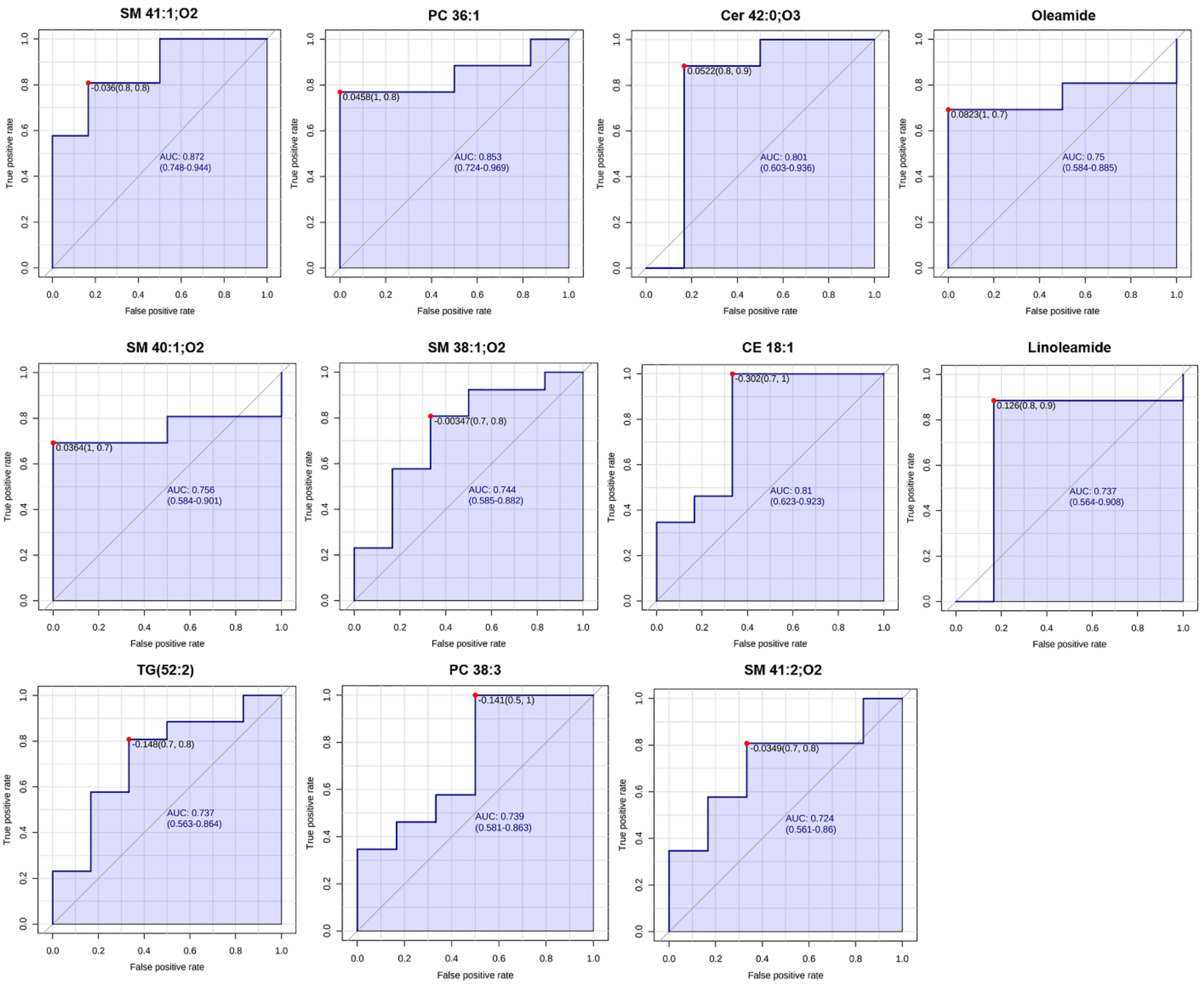

Figure S6. ROC curve of biomarker identified using serum apolar extract by HRMS spectroscopy. The sensitivity is on the y-axis, and the specificity is on the x-axis. AUC is in blue.

.

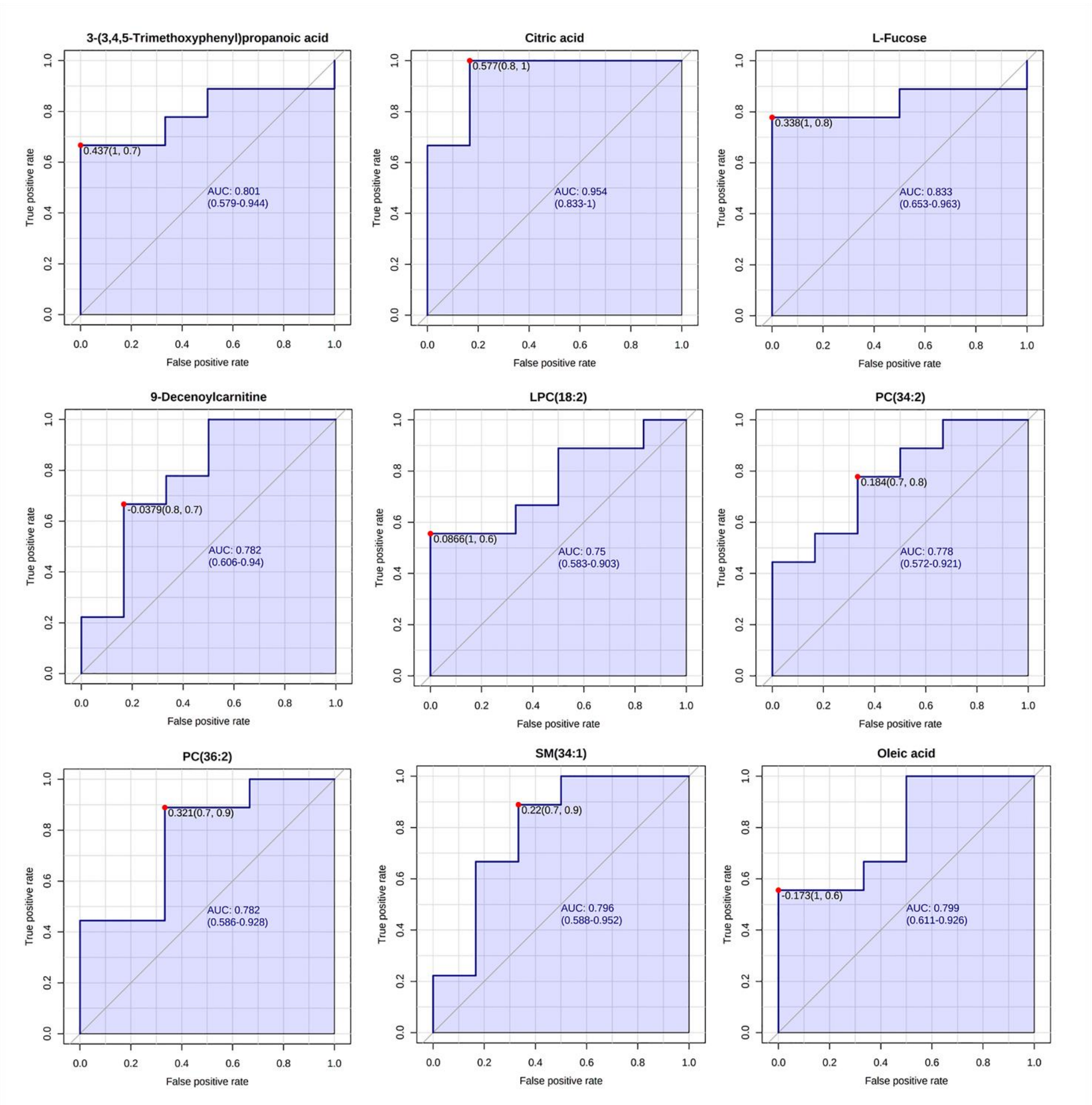

Figure S7. ROC curve of biomarker identified using serum polar extract by HRMS spectroscopy. The sensitivity is on the y-axis, and the specificity is on the x-axis. AUC is in blue

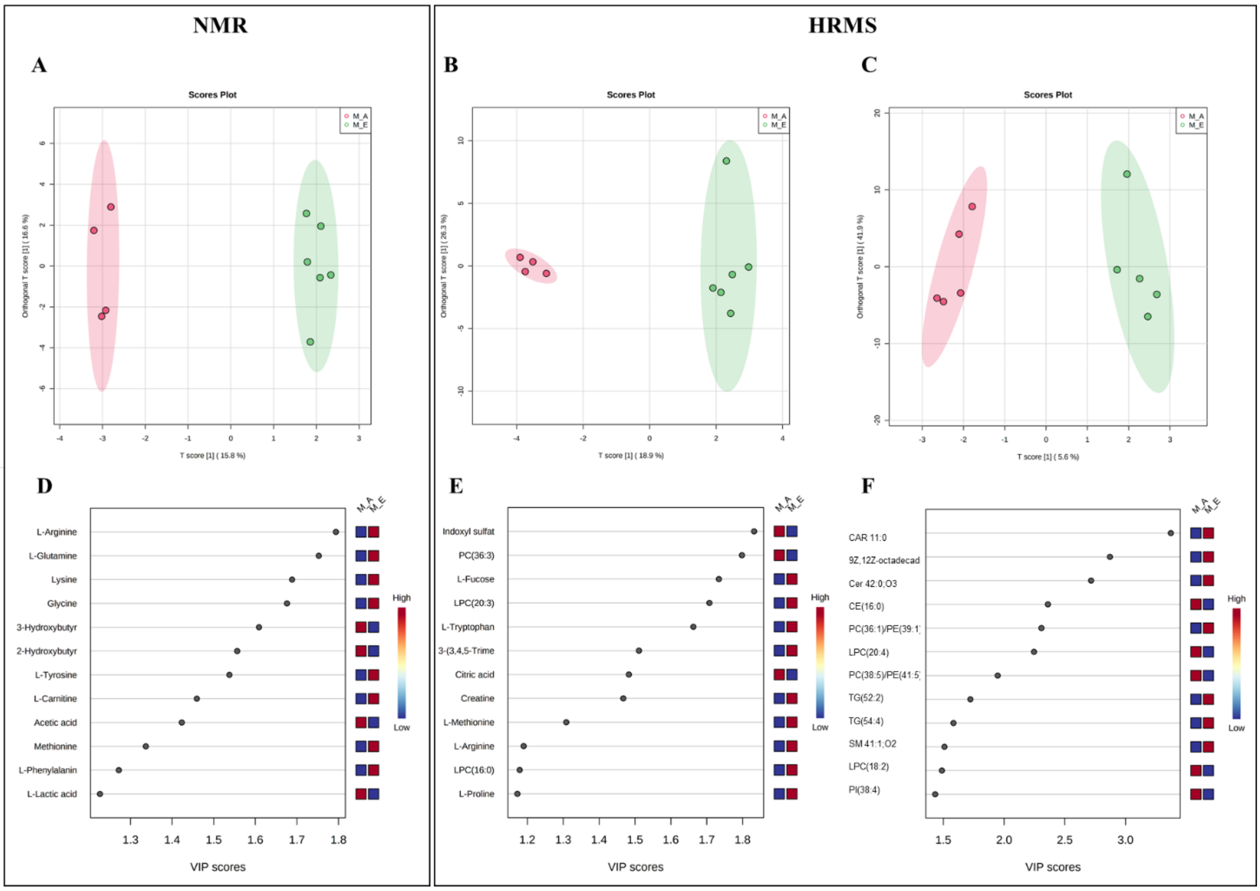

Figure S8. OPLS-DA score plot and VIP graph (A-D) for ^{1}H NMR data collected in 1D-NOESY spectra acquired at 600 MHz. Data represent the sera from 6 male ALS *early* patients (green) and 4 male ALS *advanced* patients (red). O-PLS-DA score scatter plot and VIP graph for the HRMS data collected acquired in ESI(+) and (-). Data are relative to polar and (B-E) and apolar (C-F) serum extract of 6 male ALS *early* patients (green) compared to 4 male *advanced* patients (red).
